# Psychological Distress and Resources Under Prolonged Exposure to an Environmental Threat: A Qualitative Study on Inhabitants Exposed to the *Campi Flegrei*’s Bradyseism

**DOI:** 10.3390/ejihpe16100164

**Published:** 2026-10-14

**Authors:** Annalisa Sarrecchia, Nuccio Ludovico, Alessandro Milani, Valeria Chiozza, Marino Bonaiuto

**Affiliations:** 1 Department of Psychology of Developmental and Socialization Processes, Sapienza University of Rome, 00185 Rome, Italy; annalisa.sarrecchia@uniroma1.it (A.S.);; 2 Integrated Research on Energy, Environment and Society (IREES), Energy and Sustainability Research Institute Groningen (ESRIG), University of Groningen, Nijenborgh 6, 9747 AG Groningen, The Netherlands; n.ludovico@rug.nl; 3 Faculty of Medicine, University of Modena and Reggio Emilia, 42121 Reggio Emilia, Italy; 4 CIRPA—Interuniversity Research Centre in Environmental Psychology, Sapienza University of Rome, 00185 Rome, Italy

**Keywords:** prolonged environmental threat, bradyseism, psychological distress, personal and social resources, psychological adaptation

## Abstract

Prolonged environmental threats expose communities to recurring hazard cues and uncertainty, yet psychological consequences and resources involved in adaptation remain underexplored. This study examined psychological outcomes, personal and social resources, and their co-occurrence among inhabitants exposed to bradyseism in *Campi Flegrei*, southern Italy. Fifty-two semi-structured interviews were conducted with inhabitants of the *Campi Flegrei* area. Eighteen were recruited without reference to a specific stakeholder role, whereas 34 were recruited based on their institutional, professional, or community roles. Narratives were examined through theory-driven thematic analysis. Six psychological outcomes were considered, including five distress-related negative psychological outcomes (i.e., anxiety, post-traumatic symptoms, depression, sleep disturbances, and suicidal ideation) and one adaptive psychological outcome (i.e., post-traumatic growth). Distress was classified using literature-derived categories, while the Social-psychological Antecedents to Resilience for Adaptation (SARA) classified resources by locus, specificity, and availability. Anxiety-related experiences were most frequent, followed by post-traumatic stress-related experiences, depression-related experiences, and sleep disturbances; post-traumatic growth was rare, and no segments related to suicidal ideation were identified. Risk perception, experience, acceptance, cognitive reappraisal, and place attachment were frequently identified as available resources in narratives involving distress, whereas information emerged predominantly as unavailable and available social support was identified only once in the corpus. These resources co-occurred with manifestations of distress without revealing a consistent protective pattern. The findings document the coexistence of resources and distress without establishing the direction of their relationships or a protective role. Future research should clarify resource–distress directionality and examine whether perceived agency explains why some resource constellations coexist with persistent distress, whereas others are associated with more adaptive responses under prolonged environmental threat.

## 1. Introduction

Environmental threats can substantially affect human health and psychological well-being. Exposure to adverse environmental conditions has been consistently associated with a range of psychological outcomes, including anxiety, depression, and post-traumatic stress symptoms (Bonanno et al., 2010; Chang & Bodnar, 2024; Goldmann & Galea, 2014; Norris et al., 2002). Prolonged exposure to stressful environmental conditions can also induce feelings of helplessness and a lack of control, worsening stress and anxiety (Chang & Bodnar, 2024; Lawrance et al., 2022; Seligman, 1975).

Recent decades have also witnessed an increase in the frequency and impact of many natural hazards, further emphasizing the importance of understanding how different forms of environmental threat influence psychological adaptation (Centre for Research on the Epidemiology of Disasters, 2025; Heanoy & Brown, 2024). These natural hazards are commonly distinguished as rapid-onset and slow-onset (Burrows et al., 2024; Cuzzolaro & Frighi, 1991; IPCC, 2022). Rapid-onset hazards, such as earthquakes, volcanic eruptions, and flash floods, develop suddenly and are typically characterized by a clearly identifiable impact phase. Slow-onset hazards, by contrast, progressively evolve over time through a gradual accumulation of environmental changes, often without a clearly defined onset or endpoint, and include phenomena such as prolonged droughts, sea-level rise, desertification, long-term changes in temperature and precipitation patterns, and ecosystem degradation (Burrows et al., 2024; IPCC, 2022). As already noted in the disaster literature, however, this distinction should not be considered absolute, as some hazards may combine gradual environmental change with more acute manifestations depending on their evolution and context (e.g., flooding, although generally considered a rapid-onset event, may sometimes have a gradual onset; Cuzzolaro & Frighi, 1991).

Most evidence on the psychological consequences of disasters derives from studies of rapid-onset events, in which exposure has primarily been conceptualized as a potentially traumatic experience. Research has focused mainly on post-traumatic stress, anxiety, and depression, but has also documented sleep disturbances and suicidal ideation (Goldmann & Galea, 2014; Heanoy & Brown, 2024; Kõlves et al., 2013; Norris et al., 2002). Alongside these forms of distress (i.e., negative psychological outcomes), a smaller body of research has examined post-traumatic growth (i.e., a positive psychological outcome), referring to positive changes emerging through efforts to cope with adversity (Amiri et al., 2021). Drawing on a recent systematic review of the psychological impacts of geophysical disasters (Sarrecchia et al., 2026b), the present study considers six outcome domains: anxiety, depression-related experiences, post-traumatic stress-related experiences, sleep disturbances, suicidal ideation, and post-traumatic growth. Examining these domains together captures both psychological distress and, residually, positive adaptation, providing the basis for investigating how personal and social resources co-occur with different responses to prolonged environmental threat.

Increasing attention has been devoted to contexts characterized by prolonged hazard exposure, in which individuals must live with an evolving environmental threat over extended periods, especially when changes occur slowly. In these situations, psychological distress may arise from a chronic or repeated history of environmental uncertainty, exposure to risk-related cues, disruption of everyday routines, and the continuous need to adapt to a changing environment (Burrows et al., 2024; Chang & Bodnar, 2024; Lawrance et al., 2022). Conceptual frameworks developed for rapid-onset disasters may not fully capture these psychological processes associated with chronic hazard exposure, highlighting the need for research that explicitly addresses these contexts (Ostadtaghizadeh et al., 2015).

Among geographical areas targeted by chronic slowly evolving environmental hazard, the *Campi Flegrei* are a large active volcanic caldera located immediately west of Naples and are considered among the world’s highest-risk volcanic systems because of their dense urbanization. Unlike volcanoes such as Vesuvius and Etna, they have no single prominent volcanic edifice, but comprise numerous low-elevation cones and craters. The area includes Pozzuoli, Bacoli, Monte di Procida, much of Quarto, and several western districts of Naples, with approximately 550,000 residents. It is particularly known for bradyseism, or “volcanic breathing,” characterized by recurrent ground uplift and subsidence, often accompanied by low-magnitude earthquake swarms (Galderisi & Limongi, 2024; INGV, 2021). The frequency and unpredictability of these events expose local communities to persistent uncertainty, with some periods experiencing more intense phenomena. Previous research on the 1983–1984 bradyseismic crisis documented psychological distress several years after the event, particularly among residents who had experienced more consequential forms of exposure, such as evacuation or financial loss (Bland et al., 1996, 2005). This context makes the area particularly suitable for more systematically examining psychological distress and adaptation under prolonged environmental threat.

### 1.1. The Role of Personal and Social Resources in Adaptation

Individuals and communities do not respond uniformly to environmental threats. Even under comparable exposure conditions, adaptation may vary depending on the resources available to interpret the threat, to regulate responses, and to translate risk awareness into protective action.

Adaptation has been defined as the process of adjustment through which the adverse impacts of an environmental threat can be reduced or avoided (IPCC, 2014; Van Valkengoed & Steg, 2019). Within this perspective, adaptation is not solely determined by the characteristics of the threat or by the individuals’ perceptions of risk, but it also depends on the availability and mobilization of personal and social resources that enable individuals and communities to cope with environmental demands. According to the Conservation of Resources Theory, adaptation is a dynamic process in which individuals strive to acquire, protect, and mobilize valued resources in response to environmental challenges (Hobfoll, 1989, 2001).

Recent meta-analyses have synthesized the growing body of evidence on adaptation to environmental threats to identify the social-psychological resources most consistently associated with adaptive responses across different hazard contexts. Across both climate change adaptation and disaster research, these syntheses converge in showing that adaptation is supported by a constellation of personal and social resources, including self-efficacy, outcome efficacy, social norms, attitudes, information acquisition, and risk perception, rather than by any single protective factor (Van Valkengoed & Steg, 2019; Tan et al., 2024). Further meta-analytic syntheses have shown that some of these antecedents of adaptation, in turn, are also influenced by a variety of social-psychological variables that can be attributed to personal and social resources (e.g., risk perception; Theodorou et al., 2026).

This conclusion is consistent with broader reviews of the disaster resilience literature, which identify recurring personal and social resources across resilience frameworks despite considerable variation in their conceptualization and operationalization (Ostadtaghizadeh et al., 2015; Zaman & Raihan, 2023). However, despite broad agreement on the importance of these resources, comparatively little attention has been paid to understanding how different resource domains interact, as well as to the extent to which each resource domain contributes to resilience. As noted by Ostadtaghizadeh et al. (2015), existing resilience frameworks have rarely examined these interdependencies or systematically assessed the relative contribution of different resource domains to resilience.

### 1.2. The SARA Model as an Interpretative Framework

As discussed in the previous section, the literature consistently recognizes the central role of personal and social resources in supporting adaptation to prolonged environmental threats. However, these resources have predominantly been investigated as isolated predictors within theoretical models that emphasize specific adaptive processes or individual determinants, making it more difficult to develop an integrated understanding of their overall contribution to adaptation.

To address this theoretical fragmentation, the present study adopts the Social-psychological Antecedents to Resilience for Adaptation (SARA; Sarrecchia et al., 2026a) as its interpretative framework. Building on Conservation of Resources Theory (Hobfoll, 1989, 2001), the model organizes the principal personal and social resources identified in the literature within a unified conceptual framework. The 29 resources were selected by systematizing factors associated with adaptation in published reviews and meta-analyses on climate-change adaptation and responses to natural disasters (Van Valkengoed & Steg, 2019; Tan et al., 2024).

In the present study, SARA provides a framework for organizing the resources examined in the literature according to their individual versus social nature and their degree of specificity to environmental risk.

Personal resources encompass individual characteristics, beliefs, and competencies, whereas social resources concern interpersonal, community, and broader contextual factors. Similarly, generic resources may support adaptation across contexts, whereas risk-specific resources are linked to a particular environmental threat. The resulting four domains therefore provide the organizing framework used in this study to integrate and interpret the social-psychological factors identified across the literature. The model had not been empirically examined before the present study, which represents its first exploratory application.

### 1.3. The Present Research

Although growing attention has been devoted to the psychological consequences of environmental threats, empirical evidence continues to derive predominantly from rapid-onset disasters, whereas the processes through which individuals and communities adapt to prolonged environmental threats remain comparatively underexplored (Bonanno et al., 2010; Burrows et al., 2024; Lawrance et al., 2022; Norris et al., 2002). In particular, limited attention has been devoted to examining how psychological outcomes and personal and social resources jointly emerge within the narratives of individuals living under conditions of prolonged environmental threat. Against this background, guided by the SARA framework, the present study examines 52 qualitative interviews with a heterogeneous sample from the *Campi Flegrei* area (Naples, Italy), including institutional representatives, risk-management professionals, members of local organizations, and residents of the local communities most exposed to bradyseism. Specifically, it explores how psychological outcomes and personal and social resources are described in participants’ narratives and examines their patterns of co-occurrence under conditions of prolonged environmental threat. Participants’ narratives were analyzed using a theory-driven thematic analysis to address three research questions:

RQ1 examines the main profiles of psychological outcomes emerging from the interview corpus, using a coding framework informed by a previous systematic review of the psychological health impacts associated with geophysical natural disasters (Sarrecchia et al., 2026b).

RQ2 identifies the personal and social resources, both generic and risk-specific, most frequently represented in participants’ narratives, using the SARA model (Sarrecchia et al., 2026a).

RQ3 explores the associations between psychological outcome profiles and the personal and social resources identified through the thematic analysis.

## 2. Materials and Methods

Before presenting the findings, it is important to clarify that the psychological distress categories adopted in this study (i.e., anxiety, post-traumatic symptoms, depression, sleep disturbances, and suicidal ideation) were derived from the psychological and clinical literature through a previous systematic review (Sarrecchia et al., 2026b) and were used exclusively as theoretical coding categories. Accordingly, the findings should be interpreted as a description of the themes emerging from the interview corpus and should not be understood as indicators of the presence or prevalence of specific psychopathological conditions.

Throughout the manuscript, terms such as anxiety, depression, or post-traumatic symptoms post-traumatic stress-related experiences therefore refer exclusively to interpretative categories used to organize the qualitative material.

### 2.1. Procedure

The present study was conducted to investigate psychological adaptation processes among individuals exposed to a prolonged environmental threat, namely the bradyseismic phenomenon, in the *Campi Flegrei* area (Naples, Italy).

Recruitment was preceded by a stakeholder mapping exercise designed to identify the actors most directly involved in the management, communication, response to, and everyday experience of the bradyseismic phenomenon. Based on this mapping, participants were recruited from local institutions, healthcare services, law enforcement agencies, civil society organizations, media organizations, and residents living in the *Campi Flegrei* area.

Potential participants included in the resulting purposive sample were subsequently contacted by professionals from Studio Saperessere s.r.l. (Rome, Italy), a research agency with training and experience in social research. The study was conducted within a broader contact framework with the Italian Civil Protection Department, while the scientific coordination was entirely by Sapienza University of Rome’s research team. Data collection was conducted between October and December 2025. Potential participants were contacted individually by e-mail or telephone and received a brief description of the study objectives and participation procedures. Those who agreed to participate were subsequently contacted to schedule an interview. Whenever possible, interviews were conducted in person within the *Campi Flegrei* area, allowing data to be collected in the environmental and social context in which participants experienced the bradyseismic phenomenon. When in-person interviews were not feasible, interviews were conducted remotely via videoconferencing platforms (approximately 50% of the total).

All interviews were conducted in Italian, audio-recorded with participants’ informed consent, and subsequently transcribed verbatim. Prior to analysis, all transcripts were anonymized to protect participants’ confidentiality.

The interviews were conducted in accordance with the ethical principles of the Declaration of Helsinki and with the applicable regulations on personal data protection. The study is reported in accordance with the Consolidated Criteria for Reporting Qualitative Research (COREQ; Tong et al., 2007). The completed COREQ checklist is reported in S6 of the Supplementary Materials. Before the interview, all participants received written information about the study and provided written informed consent.

### 2.2. Interview Structure

A semi-structured interview guide comprising open-ended questions and optional probes was used (see S2 of the Supplementary Materials). Questions followed a standard sequence and covered two thematic areas: (1) everyday experience of living with the bradyseismic phenomenon; (2) personal and social resources. Risk perception was not addressed through a dedicated question but emerged spontaneously in both thematic areas.

Interviews were co-conducted by faculty members, researchers, and PhD candidates from Sapienza University of Rome, all psychologists with experience in qualitative research. The interviews were conducted together with colleagues from other university teams, specifically the University of Naples “Federico II”, the University of Genoa, the University of Bari “Aldo Moro”, and another Sapienza University of Rome team of communication scientists.

Participants were invited to describe changes in their daily lives, and the resources they considered useful for coping with the situation. When appropriate, interviewers used probes to clarify or further explore issues that emerged spontaneously.

Psychological outcomes were not assessed through questions explicitly addressing symptoms; rather, references to psychological outcomes emerged spontaneously from participants’ narratives and were subsequently identified through the theory-driven thematic analysis described in Section 2.4. Personal and social resources were identified through the same analytical process.

Interviews lasted approximately 45–60 min, and each interview was attended by one to three researchers. The study was conducted within a theory-informed social-psychological perspective. The interview guide and coding framework were informed by the SARA model and by the research team’s disciplinary backgrounds in psychology and the social sciences. Transcripts were not returned to participants for comment or correction.

### 2.3. Research Sample

Participant selection was guided by a purposive sampling strategy based on stakeholder role and geographical exposure in order to capture complementary perspectives across the local social system and variation across exposure contexts.

Recruitment focused on Pozzuoli, the area most directly affected by recurrent seismic activity, and included participants from Bacoli, Bagnoli, Quarto, and other exposed districts of Naples.

The final sample comprised 52 participants, all of whom were inhabitants of the *Campi Flegrei* area, as residence in the area was an eligibility criterion. Eighteen participants were recruited as community members, whereas 34 were recruited based on their professional, institutional, or community roles, including public administration, healthcare, law enforcement, education, civil society, and religious organizations (see Table S1 of the Supplementary Materials). Twenty-five of the interviews were conducted remotely via videoconferencing platforms.

The sample comprised 32 men and 20 women. Participants were distributed across five age bands: 11 were aged 20–30 years, 17 were aged 30–40, 6 were aged 40–50, 16 were aged 50–60, and 2 were over 60 years old. Notably, all participants in the youngest age group were residents of Pozzuoli. This demographic concentration should be considered when interpreting findings involving age or place of residence, as the two characteristics may be partially confounded in the present sample.

Sample size was determined by the purposive sampling strategy and the study’s aim of including participants representing different roles and geographical contexts of exposure to bradyseism. The final sample of 52 interviews covered the participant roles and locations targeted by the recruitment strategy and provided material addressing all research questions. Formal saturation was not used as a stopping criterion. This rationale is consistent with the information-power approach, according to which sample adequacy depends on the study aim, sample specificity, theoretical grounding, quality of dialogue, and analytic strategy (Malterud et al., 2016).

### 2.4. Coding Framework and Coding Procedure

Interview transcripts were analyzed using a thematic coding framework comprising 35 predefined categories: six psychological outcomes (Sarrecchia et al., 2026b) and 29 personal and social resources (Sarrecchia et al., 2026a). The first author manually screened the 52 transcripts to identify passages concerning bradyseism that corresponded to at least one of these categories; passages unrelated to the predefined categories were excluded. The selected passages were then segmented by the first author into semantically coherent meaning units, which constituted the analytic units. A single interview could contribute multiple analytic units, and each unit could be assigned to one or more categories. This procedure yielded 164 analytic units, which formed the corpus used to calculate frequencies and Lift values. Of these, 32 were derived from participants recruited without reference to a specific stakeholder role, and 132 from participants recruited based on their institutional, professional, or community roles.

Six categories captured the psychological outcomes, including five distress-related outcomes (i.e., anxiety, post-traumatic symptoms, depression, sleep disturbances, and suicidal ideation) and one adaptive psychological outcome (i.e., post-traumatic growth). These categories were coded dichotomously as absent (0) or present (1) within the analytic unit.

The remaining 29 categories captured personal and social resources as defined in the SARA framework. They were organized into four domains arising from the intersection of the personal/social and generic/risk-specific dimensions: generic personal resources, risk-specific personal resources, generic social resources, and risk-specific social resources. Within each analytic unit, every resource category was assigned one of three mutually exclusive states: unavailable (−1), not identified (0), or available (1). A resource was coded as available (1) when the narrative indicated that it was present, accessible, or mobilized in responding to the bradyseismic phenomenon. It was coded as unavailable (−1) when the participant explicitly described its insufficiency, impairment, or inaccessibility. A value of 0 indicated that the resource was not represented in that analytic unit.

Multiple psychological outcome and resource categories could be assigned to the same analytic unit when the corresponding contents co-occurred within the segment. The Coding Manual with definitions, coding rules, and inclusion and exclusion criteria for psychological outcome categories are provided in S3 of the Supplementary Materials (see also Table S3; Lowe et al., 2019; Tedeschi & Calhoun, 2004). Corresponding definitions and criteria for resources and resource states are provided in S4 of the Supplementary Materials (Table S4a,b), with examples of their application to interview excerpts reported in Table S5.

To assess the consistency and stability of the coding framework, the primary coder and a second coder independently coded a subset of 16 analytic units using the same Coding Manual. Agreement was evaluated across all 35 categories, corresponding to 560 coding decisions (i.e., 35 code categories across 16 analytic units). Across all categories, agreement was 97.0%, with Cohen’s κ = 0.83, 95% CI [0.74, 0.90]. For the 29 resource categories (464 coding decisions), agreement was 96.3%, κ = 0.80, 95% CI [0.69, 0.89]. Across the six psychological outcome categories (96 coding decisions), agreement was 100.0%, κ = 1.00. The reliability subset contained seven positive outcome codes: anxiety (*n* = 3), post-traumatic stress-related experiences (*n* = 2), depression-related experiences (*n* = 1), and sleep disturbances (*n* = 1); no positive codes were present for suicidal ideation or post-traumatic growth.

Following the reliability assessment, coding disagreements were discussed by the coders and category boundaries were clarified where necessary. The primary coder then coded the full corpus of 164 analytic units using the refined coding framework.

After coding completion, the passages assigned to each psychological outcome category were reread comparatively to identify recurrent narrative features and to select representative quotations for the qualitative presentation of the findings.

Following the analyses addressing RQ1–RQ3, the recurrence of references to habituation to bradyseism prompted a post hoc descriptive review of the corpus. This coding included both explicit mentions of being accustomed or habituated and accounts describing this type of adaptation process to bradyseism. As this review was exploratory and conducted after completion of the primary coding process, it was not included in the assessment of intercoder agreement.

### 2.5. Data Analysis

The coding procedure described above produced a matrix in which each row corresponded to an analytic unit and each column to one of the 35 coding categories. All analyses were conducted on this matrix and were designed to describe how psychological outcomes and social-psychological resources are jointly narrated within the interview corpus. The descriptive statistics underlying the analyses addressing RQ1–RQ3 are reported in S5 of the Supplementary Materials (Tables S6–S8).

To address RQ1, we counted the analytic units in which each of the six psychological outcome categories was coded, reporting both the absolute number of coded occurrences and the proportion of analytic units in which the category appeared, together with the number of participants contributing to each (Table S6 of the Supplementary Materials). Suicidal ideation received no coded occurrence in the corpus and was therefore not included in subsequent co-occurrence analyses. These frequencies provided the quantitative background against which the narrative features of each psychological outcome were then examined qualitatively.

For RQ2, each of the 29 resource categories was decomposed into two separate indicators corresponding to the two states in which a resource could be narrated, available and unavailable, yielding 58 resource-states. For each resource-state, we recorded the number of analytic units in which it was coded and the number of participants who contributed at least one such unit (Table S7 of the Supplementary Materials). Resource-states were then aggregated into the four domains of the SARA model using indicators that reported whether at least one resource belonging to a given domain in a given state was coded within an analytic unit.

To address RQ3, we explored the relationship between psychological outcomes and resource states in participants’ accounts. We defined a local co-occurrence as the presence of two codes within the same analytic unit and cross-tabulated each resource-state with each psychological outcome. Each resource-state and psychological outcome was represented as a binary indicator at the analytic-unit level and could therefore contribute at most once to a given resource-state–outcome co-occurrence, irrespective of repeated mentions within the segment. The number of co-occurring analytic units is the primary information reported. This count, however, is affected by how often each code appears in the corpus as a whole, since a resource coded in many units will co-occur with several outcomes simply because of its frequency. Lift was therefore used alongside the count: by relating the observed co-occurrence to the co-occurrence expected from the frequency of the two codes taken separately, it indicates whether a given pairing is characteristic of that resource or largely a by-product of how often the resource occurs. The Lift score is calculated as:

$$\mathrm{Lift}=\;\frac{n_{\mathrm{ro}}}{(n_{r}n_{o})/N}$$

where *n_ro_* is the number of analytic units in which resource-state *r* and outcome *o* were both coded, *n_r_* and *n_o_* the number of units in which each was coded separately, and *N* the total number of analytic units. A value of 1 indicates that the two codes appear together about as often as their separate frequencies would predict; values above 1 indicate that they are over-represented, while values below 1 indicate that they are under-represented.

Detailed results of the co-occurrence analysis, including exact co-occurrence counts and Lift values, together with the number of unique participants contributing to each resource-outcome combination, are reported in Table S8 of the Supplementary Materials.

As a further analytical step, the configuration of resources surrounding each psychological outcome was explored. A network approach was adopted to examine which resources co-occur with a given psychological outcome. Within the network, psychological outcomes and resource-states were represented as nodes, while resource–outcome and resource–resource co-occurrences were represented as undirected ties.

The network made it possible to explore patterns of co-narration across the interview corpus, in which resources were described together and discursively anchored to a specific distress or to post-traumatic growth.

To focus the analysis on repeated and over-represented relations among nodes, ties were retained only if they met a minimum level of support. A tie was retained when the two codes co-occurred in at least two analytic units, and their Lift exceeded 1, that is, when the co-occurrence was both repeated and more frequent than the separate frequencies of the two codes would predict.

Analyses were carried out in Python 3.12 using the Matplotlib (version 3.11.1) and NetworkX (version 3.6.1) libraries.

## 3. Results

The findings are presented according to the three research questions: profiles of psychological distress and post-traumatic growth (RQ1), personal and social resources (RQ2), and their patterns of co-occurrence (RQ3). Throughout the Results, participants are identified by anonymized codes consisting of the letter P followed by a unique identification number (e.g., P4).

### 3.1. Profiles of Psychological Distress and Post-Traumatic Growth (RQ1)

**Figure 1:**
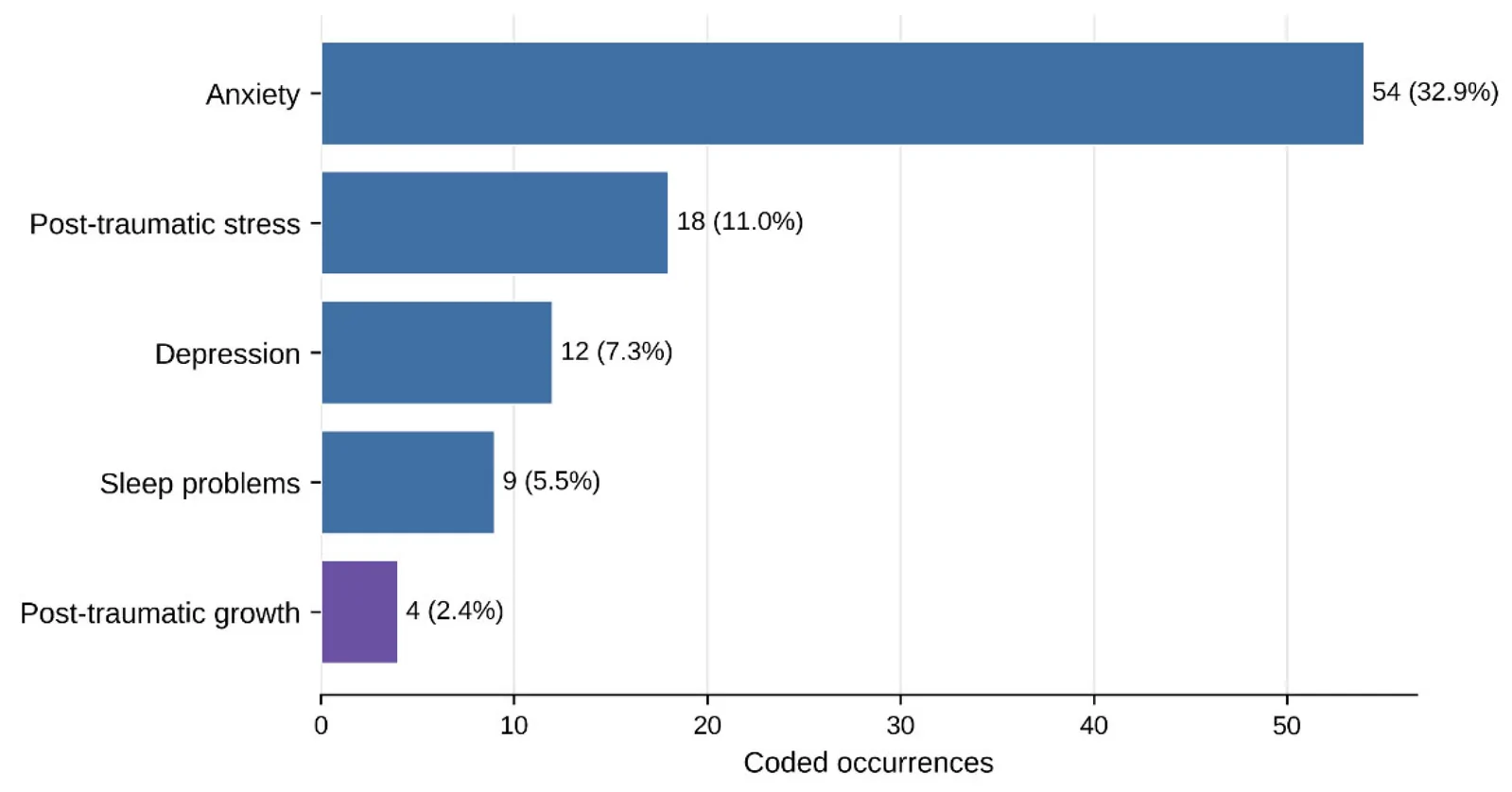
Frequency of psychological distress domains and post-traumatic growth (PTG). ***Note.*** Bars represent the number and proportion of analytic units (*N* = 164) coded for each outcome domain. Distress outcomes are shown in blue and post-traumatic growth (PTG) in purple.

Figure 1 shows the frequency of the domains identified. Anxiety was the most frequent (54 occurrences contributed by 30 participants), followed by post-traumatic stress-related experiences (18 occurrences; 14 participants), depression-related experiences (12 occurrences; 11 participants), and sleep disturbances (9 occurrences; 8 participants). Post-traumatic growth (PTG) was infrequent (4 occurrences; 4 participants), and no segments were coded as suicidal ideation (Table S6 of the Supplementary Materials).

#### 3.1.1. Anxiety

Anxiety-related narratives comprised three patterns (see Table 1): uncertainty about the evolution and consequences of bradyseism (P4, P6, P18); persistent apprehension, vulnerability, and insecurity in everyday life (P18, P31, P37); and heightened alertness to possible signs of seismic activity (P6, P8, P10, P52). Participants also reported night-time startle responses (P50) and precautionary behaviours, such as keeping an emergency bag ready or sufficient fuel in the car (P17). Anxiety therefore extended beyond individual earthquakes and remained embedded in daily routines.

#### 3.1.2. Post-Traumatic Stress-Related Experiences

Post-traumatic stress-related experiences reflected the persistence of fear and insecurity beyond individual seismic events (see Table 2). They centred on intense earthquakes that remained salient reference points (P35, P31); difficulty restoring safety and returning home after an event (P29, P30); and intense emotional reactions among children, people without previous earthquake experience, and individuals unable to leave or take protective action (P17, P36). Several participants described continuing to feel under threat between earthquakes as seismic activity repeatedly recurred.

#### 3.1.3. Depression-Related Experiences

Depression-related narratives referred primarily to demoralization, diminished hope, and resignation (see Table 3). Participants described uncertainty about their personal and territorial future (P4), perceived community decline and residents leaving their homes (P6, P31), and sadness about becoming accustomed to recurrent earthquakes (P45). These accounts also included demands for greater protection and intentions to remain in the area (P6), showing that resignation coexists with continued attachment to the territory.

#### 3.1.4. Sleep Disturbances

Sleep-related narratives described disruption across consecutive nights during seismic swarms (P6, P11, P51), changes in sleeping arrangements motivated by perceived insecurity (P28, P29), and difficulty resting because of anticipated nocturnal earthquakes (P45, P50). Some residents reportedly slept in cars or temporary accommodation and returned home only during the day (see Table 4).

#### 3.1.5. Post-Traumatic Growth

The four PTG-coded occurrences did not provide clear evidence of individual psychological growth. Instead, they reflected community-oriented meaning-making: participants reappraised the volcanic characteristics of the *Campi Flegrei* as elements of place identity and potential sources of cultural learning (P7, P22), and described adversity as an opportunity to promote the territory (P21). Participants describe these positive reinterpretations as contingent on adequate prevention and safety measures, linking positive meaning-making to the availability of social and institutional protection (see Table 5).

### 3.2. Personal and Social Resource Profiles (RQ2)

**Figure 2:**
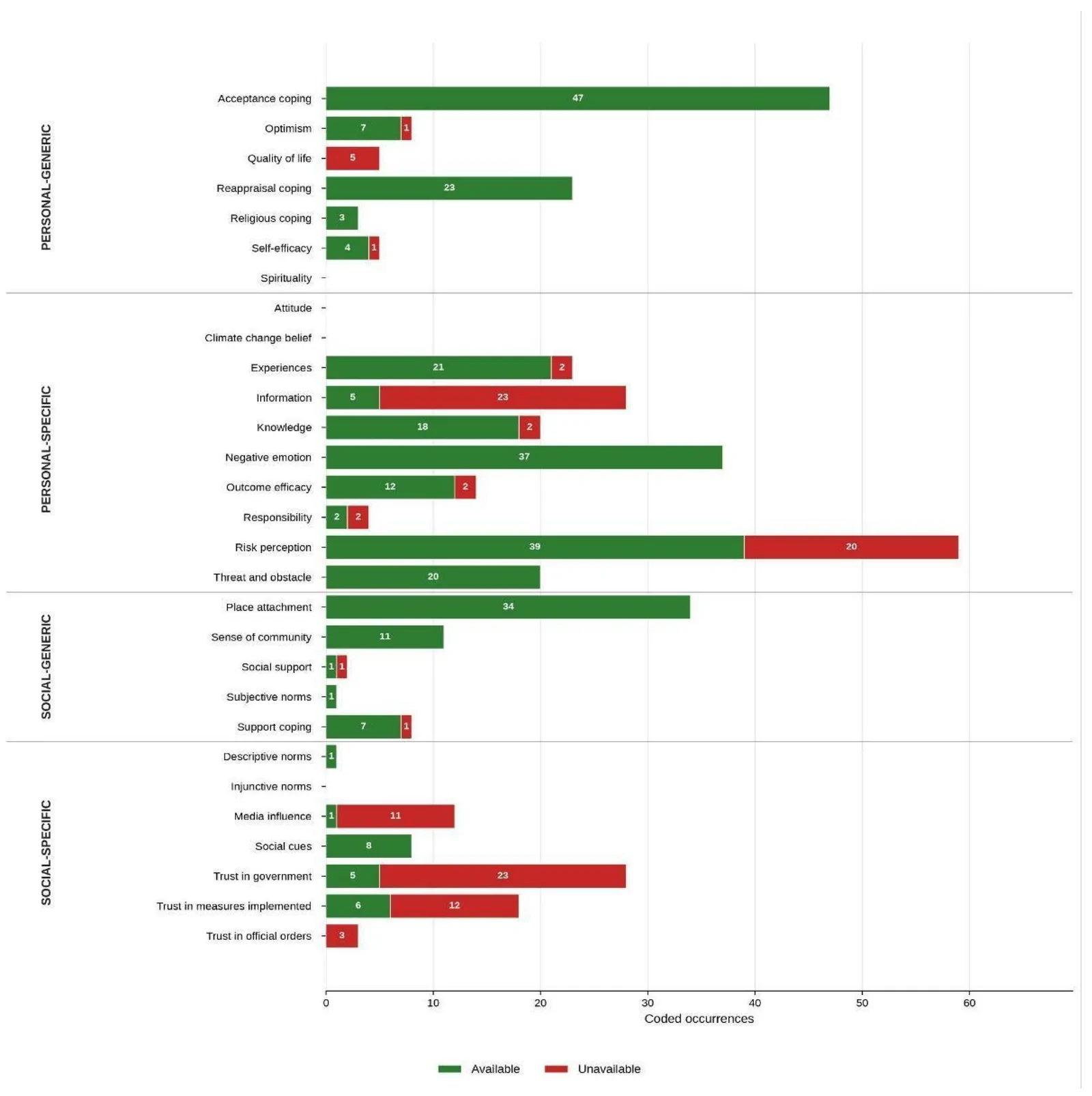
Resource-state coded occurrence profile by SARA category. ***Note.*** Bars represent the number of coded occurrences for each resource construct, grouped by SARA category: PER-GEN (personal-generic), PER-SPEC (personal-specific), SOC-GEN (social-generic), and SOC-SPEC (social-specific). Each occurrence was coded as reflecting either an available (green) or unavailable (red) resource state.

Personal and social resources emerge across all four SARA domains (Figure 2), with available resource states occurring more frequently overall than unavailable states. Analytic-unit and participant-level frequencies for each resource-state are reported in Table S7 of the Supplementary Materials.

Generic personal resources centre primarily on acceptance and cognitive reappraisal, whereas optimism, self-efficacy, and religious coping occur less frequently. Reduced quality of life represents the main unavailable resource in this domain. Threat-specific personal resources constitute the most prominent domain. Risk perception and awareness of negative emotions are the most frequent, followed by previous experience, perceived threat characteristics, and knowledge. Lack of information is the most frequently reported unavailable resource, while risk perception occurs in both available and unavailable states.

Among generic social resources, place attachment predominates, followed by sense of community, while unavailable states remain rare. Threat-specific social resources show the opposite pattern: unavailable states concern primarily trust in government, trust in the measures implemented, media influence, and trust in official orders.

Overall, personal resources were coded more frequently than social ones. The distribution of available and unavailable states also differs across the two threat-specific domains. At the personal level, awareness of negative emotions, threat and obstacle perception, risk perception, previous experience, knowledge, and outcome efficacy were coded predominantly as available, whereas information was coded more frequently as unavailable. At the social level, threat-specific resources were coded predominantly as unavailable, particularly those concerning trust and media influence. Four predefined resource categories, namely spirituality, attitude, climate change belief, and injunctive norms, were not identified in any analytic unit.

### 3.3. Resource–Psychological Outcome Co-Occurrences (RQ3)

**Figure 3:**
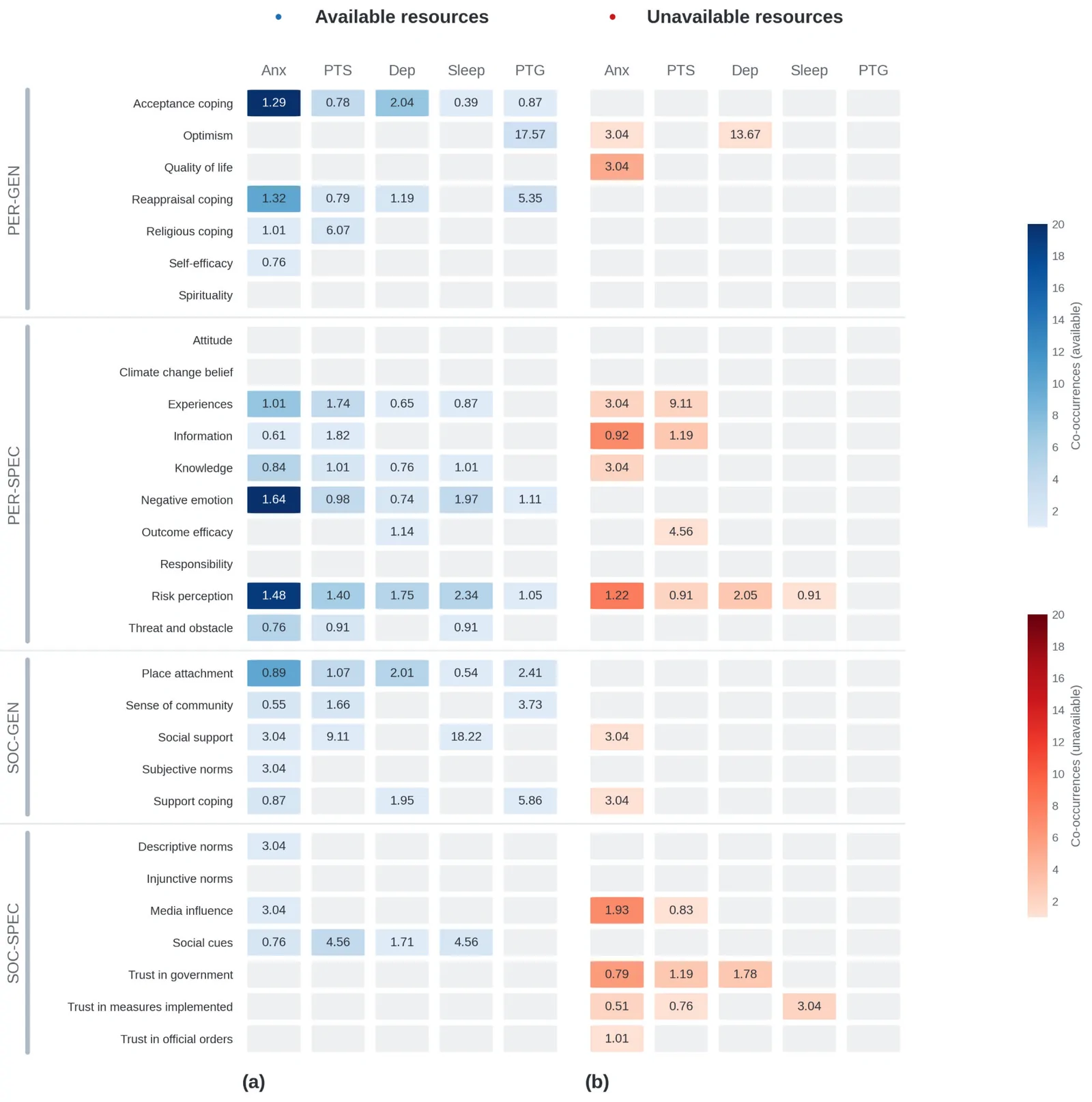
Co-occurrences between resource-states and psychological outcomes by resource availability. ***Note.*** Each panel is a resource × outcome matrix computed at the analytic unit level. Panel (**a**) shows available resource-states (blue) and panel (**b**) shows unavailable resource-states (red). Rows are the 29 resources of the SARA framework, grouped by dimension (PER-GEN = personal-generic, PER-SPEC = personal-specific, SOC-GEN = social-generic, SOC-SPEC = social-specific); columns are the psychological outcomes (Anx = anxiety, PTS = post-traumatic stress-related experiences, Dep = depression-related experiences, Sleep = sleep disturbances, PTG = post-traumatic growth). Suicidal ideation is not shown. Cell colour encodes co-occurrence count. Cell labels report the Lift score. Grey cells indicate no observed co-occurrence.

Co-occurrences between psychological outcome profiles and resources were examined at the SARA-category level and at the level of individual resources (Figure 3). A co-occurrence indicates that two codes appear within the same analytic unit and does not establish a causal relationship. Lift values indicate whether a resource is overrepresented with a given profile relative to its overall frequency in the corpus. Exact analytic-unit and participant counts and Lift values underlying Figure 3 are reported in Table S8 of the Supplementary Materials.

The profiles show distinct configurations. Anxiety presents the broadest constellation, including available and unavailable personal resources and unavailable social resources related to risk communication. Depression-related experiences co-occur most frequently with available threat-specific personal resources, followed by generic personal and generic social resources. Sleep difficulties are also concentrated around threat-specific personal resources, whereas post-traumatic stress-related experiences co-occur with both personal and social resources. Post-traumatic growth presents the most circumscribed configuration, mainly co-occurring with generic personal and social resources.

Lift analyses showed that co-occurrence frequency and over-representation did not always coincide (Figure 3a,b).

For anxiety, acceptance, awareness of negative emotions, and risk perception showed the highest co-occurrence counts, but unavailable media influence (*n* = 7; *lift* = 1.93) and unavailable quality of life (*n* = 5; *lift* = 3.04) were more concentrated in anxiety-related units relative to their overall frequency. Depression-related experiences showed a more specific configuration involving acceptance (*n* = 7; *lift* = 2.04), place attachment (*n* = 5; lift = 2.01), and unavailable risk perception (*n* = 3; *lift* = 2.05). For post-traumatic stress-related experiences, social cues were strongly over-represented (*n* = 4; *lift* = 4.56), while larger lift values based on only two co-occurrences were interpreted cautiously. At the category level, post-traumatic growth was over-represented with available generic personal resources (*lift* = 2.73), whereas sleep disturbances showed more moderate over-representation with available threat-specific personal and social resources (*lift* = 1.26 and 1.92, respectively). Lift values describe the specificity of these co-occurrences relative to corpus-wide frequencies and do not indicate direction or causality.

Suicidal ideation was not included in the lift analysis because no textual segments were coded for this outcome.

#### 3.3.1. Resource Configurations Associated with Anxiety

Anxiety shows the broadest resource constellation. Acceptance, cognitive reappraisal, experience, and risk perception occur in narratives in which fear and uncertainty remain present. Participants describe becoming more familiar with earthquakes, recognizing their signals, and knowing how to respond, while distinguishing this practical familiarity from emotional habituation:

“*I am used to it in the sense that I am trained: I can recognize the earthquake, so I am prepared in the sense that I know how to behave. But you never really get used to it. I believe this creates uncertainty about the future and makes it more difficult to plan everyday life.*”

(P18)

Experience therefore co-occurs with greater knowledge of how to respond, but also with continuing uncertainty and difficulty planning everyday life. Other narratives describe acceptance and cognitive reappraisal alongside changes in reactions to earthquakes, including remaining at home or responding only above magnitudes previously considered sufficient to leave the building (P35, P17).

Unavailable media influence shows seven direct co-occurrences with anxiety and is overrepresented relative to its frequency in the corpus (lift = 1.93; Figure 3). In these units, references to alarmist social-media content occur alongside panic, heightened alertness, and exhaustion:

“*At a certain point, the Facebook groups generate an overwhelming level of alarmism that sends people into a state of panic. [...] We encourage people to follow Civil Protection channels*.”

(P8)

Anxiety thus co-occurs both with personal resources used to interpret and respond to earthquakes and with perceived deficiencies in the information environment.

#### 3.3.2. Resource Configurations Associated with Depression-Related Experiences

Depression-related experiences co-occur most frequently with available threat-specific personal resources (8 of 12 units; 66.7%), generic personal resources (7; 58.3%), and generic social resources (6; 50%). Acceptance is the most frequent individual resource, followed by place attachment and available risk perception. Unavailable risk perception and unavailable trust in government also occur within this configuration (Figure 3).

Acceptance and altered thresholds for responding to earthquakes appear alongside demoralization and representations of territorial decline:

*“Anything below 3.5 no longer really counts... Yet, it is a depressed situation, as though we had been assigned this inevitable fate [...] businesses closing, people leaving their homes to move elsewhere.”*

(P31)

The shift in response thresholds also emerges in accounts of everyday life. One participant describes earthquakes as “part of everyday life,” reports sleeping through an event, and discourages her grandmother from leaving the house: “What are we supposed to do anyway? If an earthquake comes, are we really going downstairs? It is ten degrees outside; that would be worse for you” (P35). Following displacement, another participant remains in the area by moving into her grandmother’s building, which she describes as having “some very serious cracks” but as “more resistant than mine.” She also reports leaving the building only “from magnitude 4 onwards,” compared with magnitude 2.5 previously (P34).

These accounts document changes in everyday routines and higher thresholds for protective action. Within the same analytic units, acceptance, cognitive reappraisal, risk perception, and place attachment co-occur with resignation, diminished hope, and perceived limitations on action. Again, the co-occurrence analysis does not necessarily establish any direction of these relationships.

#### 3.3.3. Resource Configurations Associated with Sleep Difficulties

Sleep difficulties co-occurred with available threat-specific personal resources in 7 of 9 analytic units (77.8%). Available risk perception was the most frequent individual resource (*n* = 5; *Lift* = 2.34), followed by awareness of negative emotions (*n* = 4; *Lift* = 1.97). Available social cues occurred in two units and were overrepresented relative to their corpus-wide frequency (*Lift* = 4.56). Available social support yielded the highest Lift (18.22), but this value was based on a single co-occurrence contributed by one participant and was therefore not interpreted as characteristic of the sleep-related profile (Figure 3; Table S8 of the Supplementary Materials).

Participants locate sleep disruption within the alternation between quiet periods and recurrent earthquake swarms. They describe consecutive nights without sleep, frequent night-time startle responses, sleeping away from home, and keeping emergency equipment ready (P50, P51). Accounts in which sleep disruption co-occurred with risk perception and negative emotional responses linked altered sleeping patterns directly to periods of intense seismic activity and fear:

*“... nothing happens for a week, and then you do not sleep at night for four days.”*

(P51)

Sleep difficulties thus emerge primarily in narratives combining perceived seismic threat with negative emotional responses and changes in sleeping arrangements.

#### 3.3.4. Resource Configurations Associated with Post-Traumatic Stress-Related Experiences

Post-traumatic stress-related experiences co-occur most frequently with available threat-specific personal resources (12 of 18 units; 66.7%), followed by unavailable threat-specific personal resources and available generic social resources (7 units each; 38.9%). Risk perception is the most frequent individual resource, while experience, place attachment, and social cues each occur four times. Unavailable information and unavailable trust in government each show three co-occurrences (Figure 3). Perceived lack of information and protection co-occur with intense fear reactions:

*“Information was completely lacking... We are not protected at all... I saw children from our parish vomiting with fear.”*

(P36)

Repeated exposure co-occurs with detailed knowledge of earthquake signals. One participant reports, “I could hear the rumble approaching from a distance and knew that the earthquake was coming,” and notes that residents “can estimate” the magnitude before its official announcement. However, this familiarity coexists with a lack of operational guidance: “If a real emergency occurs, we do not know where to go” (P33). Experience shows different configurations across participants: lower-magnitude earthquakes are sometimes incorporated into everyday routines, whereas previous crises are recalled alongside immediate flight responses and difficulty tolerating new events (P43).

Acceptance and cognitive reappraisal also co-occur with persistent alertness, expressed through detailed memories of the strongest earthquakes and reactions to minor or ambiguous vibrations. Participants who describe themselves as accustomed or adapted recall the time, location, magnitude, and sensory characteristics of specific events (P17, P35). One participant states that “you simply can’t tolerate even the slightest tremor” (P6), while another reports that “even when a truck passes by, the first reaction is fear,” as vibrations produced by heavy vehicles lead residents to worry that “it is another earthquake” (P52). These accounts show that reported adaptation coexists with continued sensitivity to environmental vibrations and immediate fear responses to stimuli resembling an earthquake.

#### 3.3.5. Resource Configurations Associated with Post-Traumatic Growth

The four units coded for post-traumatic growth all co-occur with available generic personal resources. Optimism and cognitive reappraisal each occur three times, while place attachment occurs twice (Figure 3). Rather than describing individual psychological transformation, participants reinterpret the volcanic characteristics of the *Campi Flegrei* as sources of knowledge, identity, and possible cultural enhancement:

*“We always try to turn difficulty into an opportunity. [...] Highlighting the volcanic and sulphurous features, and therefore the distinctiveness of a place such as the Campi Flegrei, could even turn it into a teacher, provided that this is embedded within a framework of prevention and safety.”*

(P22)

Hope and positive reinterpretation coexist with explicit references to suffering and danger (P24). Participants also describe the possibility of attributing cultural value to the experience of bradyseism only when the territory is protected and communicates safety (P7). These occurrences link positive meaning-making and place attachment to the availability of social and institutional protection.

**Figure 4:**
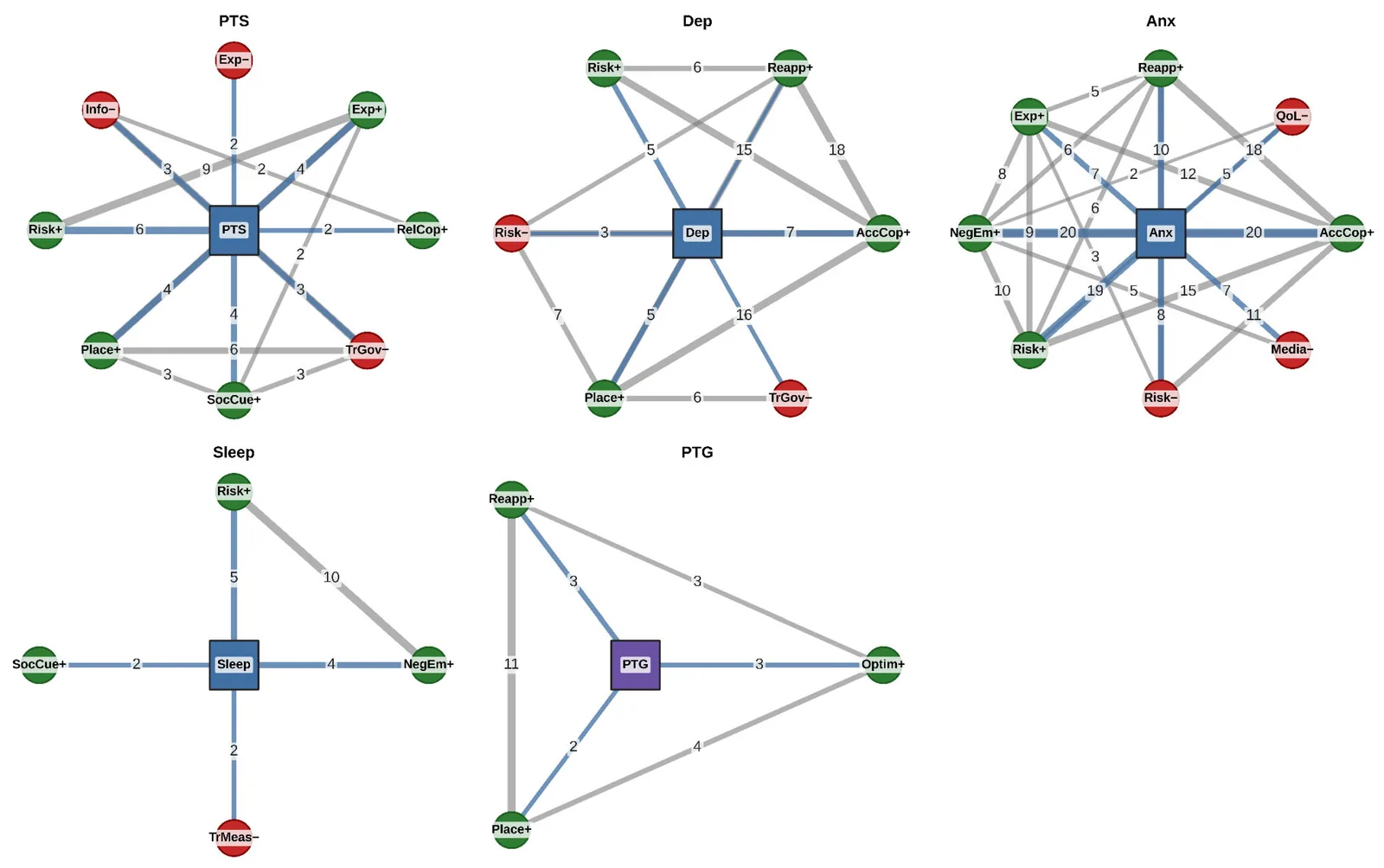
Resource configurations associated with psychological outcomes. ***Note.*** Each panel is an ego-network for one psychological outcome, shown as the central square node: PTS (post-traumatic stress), Dep (depression), Anx (anxiety), Sleep (sleep disturbance), and PTG (post-traumatic growth); distress outcomes are shown in blue and the positive outcome (PTG) in purple. Surrounding circular nodes are resources that co-occurred with that outcome. Node colour and suffix denote the coded resource state: green with a “+” indicates an available resource, red with a “−” an unavailable one (a resource may appear in both states within a panel). Edge labels give the co-occurrence frequency between two codes, and edge width is proportional to this weight. Blue edges show resource–outcome co-occurrences within the analytic units coded for the outcome; grey edges show resource–resource co-occurrences calculated across the full analytic corpus. Only edges with at least two observed co-occurrences and *Lift* > 1 are displayed.

Figure 4 brings together the resource configurations associated with each psychological outcome. Anxiety shows the broadest configuration, centred on available acceptance, awareness of negative emotions, risk perception, cognitive reappraisal, and experience, alongside unavailable risk perception, media influence, and quality of life. The post-traumatic stress configuration combines available risk perception, experience, place attachment, and social cues with unavailable information and trust in government. Depression-related experiences show a more circumscribed configuration involving available acceptance, risk perception, and place attachment, together with unavailable risk perception and trust in government. Sleep disturbances are linked to a smaller set comprising available risk perception, awareness of negative emotions, and social cues, as well as unavailable trust in protective measures. Post-traumatic growth presents the most circumscribed configuration, involving available cognitive reappraisal, optimism, and place attachment. The corpus-wide resource–resource connections further show that acceptance, cognitive reappraisal, place attachment, and risk perception form a recurrent interconnected set, whereas cognitive reappraisal, optimism, and place attachment form the compact set displayed in the post-traumatic growth panel. The grey edges contextualize these outcome–resource configurations by showing which of the displayed resources were also frequently co-narrated across the full corpus.

### 3.4. Self-Reported Habituation

Habituation was not included a priori in the codebook and was therefore not treated as a formal coding category. However, its recurrence in participants’ narratives prompted a post hoc descriptive review of explicit references to becoming accustomed and accounts describing related changes in responses to bradyseism.

A post hoc descriptive review identified habituation-related content in 15 analytic units from 15 participants. Twelve accounts explicitly used habituation-related terms: eleven affirmatively described becoming accustomed or habituated, while one account (P2) explicitly denied that the population had become accustomed to bradyseism. The remaining three accounts described changes in responses without using habituation-related terms. The affirmative and implicit accounts described reduced reactions over time (P27, P40), the incorporation of lower-magnitude earthquakes into everyday life (P16, P24), higher thresholds for interrupting activities or leaving buildings (P31, P33), and greater knowledge of how to respond (P17, P18, P45). Further details are reported in Table S2 of the Supplementary Materials.

## 4. Discussion

The present study examined the psychological outcome profiles and configurations of personal and social resources emerging from narratives of living with bradyseism in the *Campi Flegrei* area. Overall, the findings show that prolonged exposure to the phenomenon is described through the coexistence of uncertainty, vigilance, the reorganization of everyday life, and increasing familiarity with the threat. Resources do not emerge as isolated factors or elements necessarily associated with the absence of distress, but rather as constellations that take on different meanings across narrative profiles.

### 4.1. Psychological Distress and Post-Traumatic Growth Under Prolonged Threat (RQ1)

Anxiety is the most frequently coded domain, and it is characterized by concern, vulnerability, uncertainty about the future, and persistent attention to noises and vibrations. This finding is consistent with the disaster literature, which identifies anxiety and non-specific distress among the most common psychological outcomes of exposure to adverse natural events (Goldmann & Galea, 2014; Norris et al., 2002). In the context of bradyseism, however, anxiety is not exclusively related to an event that has already ended, but also to the anticipation of further earthquakes and uncertainty regarding the evolution of the phenomenon. The gradual nature of ground uplift is compounded by acute and recurrent seismic activity, making the distinction among preparedness, response, and recovery less clearly defined. These findings are therefore consistent with the literature on prolonged environmental threats, according to which persistent uncertainty and the disruption of conditions of safety may operate as ongoing stressors (Burrows et al., 2024; Lawrance et al., 2022).

Sleep disturbances and narratives concerning post-traumatic stress symptoms also reflect this temporality. Sleep is disrupted not only by individual earthquakes but also by the unpredictable alternation between periods of relative calm and earthquake swarms. At the same time, detailed memories of the most intense events coexist with the anticipation of further earthquakes and of difficulty in restoring a stable sense of safety. Previous research has extensively documented the persistence of anxiety, sleep disturbances, and post-traumatic stress symptoms following earthquakes, particularly in the presence of high levels of exposure, housing damage, and displacement (Goldmann & Galea, 2014; Norris et al., 2002).

Narratives concerning depressive symptoms predominantly took the form of demoralization and resignation rather than descriptions of individual clinical symptoms. They are concerned about difficulty envisioning the future, economic deterioration, business closures, and the possible abandonment of the territory. This finding confirms the importance of secondary stressors and the “chronic problems of living” previously identified in disaster research (Norris et al., 2002). In the *Campi Flegrei* context, distress therefore appears to be connected not only to fear of earthquakes, but also to the progressive erosion of economic and residential predictability and of the capacity to plan for the future.

Finally, references to post-traumatic growth were limited and predominantly oriented towards the collective dimension. Optimism, cognitive reappraisal, and place attachment appeared in the reinterpretation of the geological characteristics of the *Campi Flegrei* as elements of identity and potential cultural enhancement. This finding departs from an exclusively individual conception of post-traumatic growth and is consistent with research distinguishing individual, community, and societal growth following disasters (Wlodarczyk et al., 2017). In the present data, however, the positive reappraisal of the phenomenon remained explicitly conditional upon prevention and safety, confirming that the attribution of positive meaning does not imply denial of the threat.

No narrative content attributable to suicidal ideation emerged from the interviews; however, this finding indicates only the absence of evidence in the narratives analyzed and should not be interpreted as evidence of its absence in the broader population.

### 4.2. Personal and Social Resources Across the SARA Quadrants (RQ2)

With respect to RQ2, resources emerged across all four SARA quadrants (Sarrecchia et al., 2026a). Thus, at the sample level, participants reported generic and threat-specific resources located at both the personal and social levels, showing that the framework allowed the observed resource states to be mapped across the four predefined quadrants. However, these resources were not uniformly distributed across individuals: participants typically referred to different combinations of resources spanning one or more quadrants, rather than to the complete set. The following discussion focuses on the resources that were most recurrent and interpretatively salient across the narratives, with particular attention to their context-dependent and potentially ambivalent meanings.

A particularly salient finding concerns risk perception, recognized in the literature as a resource relevant to adaptation to natural hazards (Bonaiuto et al., 2016; Theodorou et al., 2026; Van Valkengoed & Steg, 2019). In the present data, risk perception accompanies the ability to recognize earthquakes, to assess building vulnerability, and to guide preparedness behaviors. At the same time, it also occurs in narratives concerning anxiety, sleep disturbances, and post-traumatic stress symptoms, taking different forms. On the one hand, repeated exposure was associated with a progressive recalibration of personal response thresholds, whereby earthquakes previously considered relevant became progressively normalized. On the other hand, persistent sensitivity to environmental cues emerges, extending to the interpretation of noises and vibrations caused by passing vehicles as possible signs of another earthquake. Rather than indicating either accurate or distorted risk perception, these findings describe different ways of appraising the threat that developed over the course of prolonged exposure.

The persistent attribution of ambiguous noises and vibrations to possible seismic activity may suggest heightened sensitivity to threat-related cues and a possible state of hypervigilance. However, the cross-sectional narrative data do not allow this pattern to be interpreted as a process of sensitization in a strict behavioral sense or as evidence of a clinical condition.

Place attachment shows a similar ambivalence. It is reflected in participants’ enduring ties to the territory, their intention to remain within the community, and their recognition of the *Campi Flegrei* as a source of collective identity and value. However, it also co-occurs with difficulties leaving one’s home, employment, and social networks, as well as with short-distance residential moves that keep people close to the area perceived as exposed. This finding is consistent with research documenting both positive and negative associations between place attachment, risk perception, and adaptive behaviours (Bonaiuto et al., 2016). These narratives also resonate with the concept of solastalgia: the distress experienced when a loved home or territory no longer provides solace, despite the person remaining in that environment (Albrecht, 2005; Stancu et al., 2025). In the *Campi Flegrei*, residents describe not only the difficulty of leaving, but also the loss of comfort associated with remaining in a familiar place that has become a source of recurrent threat. Place attachment may therefore sustain territorial continuity while intensifying the distress associated with this altered relationship with home. As solastalgia was not directly assessed, it is used here as an interpretative lens rather than an identified condition.

Information and media influence emerge predominantly in their unavailable states under configurations associated with anxiety and post-traumatic stress-related experiences. Although experience enabled some participants to recognize earthquake rumbles or estimate their magnitude, this familiarity does not necessarily coincide with knowing where to go or how to act during an emergency. Experience, therefore, helps participants interpret signals of seismic activity, but does not replace the need for clear, trusted, and actionable information.

The role of media influence can be interpreted through the Social Amplification of Risk Framework, which describes how risk signals are processed by media and interpersonal networks acting as “amplification stations” that can increase or attenuate their salience and shape public responses (Kasperson et al., 1988). In participants’ narratives, Facebook groups perceived as alarmist were described alongside panic and heightened alertness, whereas Civil Protection channels were regarded as more reliable. These findings do not establish that social media amplifies anxiety, but show that the perceived availability of information depends on its source, credibility, and capacity to support action. Risk communication therefore emerges not merely as the transmission of information, but as a social resource whose relevance depends on how risk signals are selected, circulated, and translated into operational guidance. The source of information and the channel through which it is communicated therefore shape its perceived trustworthiness and legitimacy, influencing whether risk communication supports coping and protective action or contributes to distress.

### 4.3. Cross-Quadrant Resource–Psychological Outcome Constellations (RQ3)

Consistent with Conservation of Resources Theory, resources tend to form constellations, and their meaning depends on the personal, social, and institutional conditions within which they are mobilized (Hobfoll, 1989, 2001, 2012). The resource–psychological outcome constellations identified for RQ3 are predominantly trans-quadrant. At the aggregate profile level, anxiety, post-traumatic stress-related experiences, depression-related experiences, and sleep disturbances involve resources from all four SARA quadrants, whereas post-traumatic growth involves three quadrants and does not include threat-specific social resources. However, the number and frequency of individual resources differ across profiles: anxiety shows the broadest constellation, whereas the other profiles involve more circumscribed sets of resources. These aggregate configurations do not imply that resources from all represented quadrants co-occurred within every individual analytic unit. The predominantly cross-quadrant structure is consistent with reviews emphasizing the multidimensional nature of disaster adaptation and the need to consider both generic and threat-specific resources operating at the individual, community, and institutional levels jointly (Ostadtaghizadeh et al., 2015). Overall, the findings do not provide sufficient evidence to establish a buffering or compensatory function of resources. In particular, experience, acceptance, and cognitive reappraisal occurred in segments in which participants explicitly compared different ways of responding to the threat: a less disorganized reaction, a greater capacity to understand earthquakes and guide behaviour, or fear described as less intense than during the initial stages of exposure. These narrative comparisons suggest that such resources may contribute to different ways of interpreting and responding to the threat. However, the data do not show that the absence of one resource was compensated for by the availability of another, nor do the observed co-occurrences establish that these resources reduced distress. Their potential buffering or compensatory role therefore remains a hypothesis to be tested through longitudinal and quantitative research.

Experience and acceptance occur across multiple profiles but do not show a uniform relationship with distress. Experience co-occurs with the ability to recognize earthquakes and guide protective responses, as well as recalibrated response thresholds, persistent vigilance, and difficulty planning for the future. Similarly, acceptance allows the phenomenon to be incorporated into everyday life while coexisting with anxiety, sleep disturbances, and resignation. These findings distinguish behavioural familiarity from habituation and identify agency, the capacity to influence one’s circumstances through intentional action, as a key dimension of these configurations (Bandura, 2001). In some narratives, experience and acceptance co-occur with preparedness and the reorganization of everyday routines. In others, they coexist with limited perceived control and statements such as “What can we do anyway?”. This latter pattern may be consistent with learned helplessness, where repeated uncontrollable events reduce expectations that one’s actions can affect outcomes (Maier & Seligman, 2016). Given the qualitative and non-directional nature of the analysis, learned helplessness remains an interpretative hypothesis, and the study cannot determine whether these resources precede psychological outcomes, arise from it, or are mobilized in response to it. The present qualitative data show that resources co-occur with both PTG-related narratives and distress-related narratives. Although limited, PTG-related narratives involved available resources such as cognitive reappraisal, optimism, and place attachment. These patterns indicate that resources may be narrated in relation to positive meaning-making while also occurring in accounts of distress. Thus, PTG-related narratives and positive meaning-making do not imply the absence of distress. Instead, the present findings suggest that resources may be involved in adaptive ways of interpreting and responding to the threat while coexisting with distress-related narratives. Furthermore, emotional responses may help explain the processes through which personal and social resources are activated within contexts of chronic hazard exposure. Fear, the most recurrent emotion in participants’ narratives, occurs alongside distress and persistent alertness, as well as monitoring of environmental cues and protective action. This dual pattern is consistent with the evolutionary function of fear as a conserved defensive system that detects threats and prepares the organism to respond (LeDoux, 2012; Öhman & Mineka, 2001). Fear should therefore not be treated solely as an indicator of distress or equated with panic: evidence from the Umbria–Marche earthquake indicates that it can coexist with organized responses such as evacuation, information seeking, affiliation, and protection of others (Prati et al., 2011). These findings raise the hypothesis that fear may influence whether resources sustain agency: when viable responses are perceived, it may help to mobilize available resources into protective action; when control and response options are limited, repeated alarm may instead accompany vigilance and helplessness.

A related exploratory observation concerns self-reported habituation. Participants described reduced behavioural reactivity, recalibrated thresholds for responding to earthquakes, and greater knowledge of how to respond. These accounts suggest reduced behavioural responsiveness to recurrent earthquakes but do not establish habituation in its technical sense. Habituation is response-specific, and the present data do not indicate whether emotional or physiological responses changed in parallel (Grissom & Bhatnagar, 2009; Rankin et al., 2009). Future research should examine whether self-reported habituation reflects adaptive calibration of responses, under-response to threat, or different processes across individuals and levels of earthquake intensity.

Given its exploratory nature, the study provides qualitative support for the relevance of resources previously identified in the literature and documents configurations in which resources and psychological outcomes co-occur. These findings provide a foundation for future quantitative research to examine which generic and threat-specific personal and social resources are associated with lower distress and higher PTG, whether they may attenuate the impact of prolonged threat exposure, and whether they operate as single resources or through specific combinations of resources.

### 4.4. Study Strengths

This study has three main strengths. First, it examines bradyseism as a prolonged and recurrent environmental threat, a context less frequently investigated than discrete post-disaster settings (Lawrance et al., 2022; Oppermann et al., 2021). Second, purposive recruitment included inhabitants with community, professional, and institutional roles, providing complementary perspectives on everyday experience and risk management. Third, psychological outcomes and available and unavailable personal and social resources were examined within the same narrative units using an explicit coding framework, a detailed codebook, and an assessment of intercoder reliability. This approach enabled a systematic description of resource–outcome co-occurrences while preserving their narrative context.

## 5. Practical Implications

The findings suggest four complementary areas for practice, although the exploratory and non-directional nature of the analysis requires that these implications be further investigated and validated.

### 5.1. Risk Communication and Operational Guidance

The findings highlight the importance participants attributed to comprehensible, consistent, and actionable information. Institutional communication could therefore complement updates on the phenomenon with guidance on protective behaviours, evacuation procedures, and available services. In particular, the co-occurrence between anxiety and unavailable media influence identifies risk communication as a potential area for intervention. Communication strategies should therefore distinguish the transmission of scientific updates from the provision of operational guidance, as well as ensuring that information is delivered through recognizable and trusted institutional channels (IASC, 2021; WHO, 2019).

### 5.2. Profile-Sensitive Social-Psychological Support

The different manifestations of distress identified in the narratives suggest the need for differentiated forms of social-psychological support. Persistent vigilance and sensitivity to environmental cues may require different interventions from those addressing sleep disruption, memories of intense earthquakes, or from demoralization related to economic decline and uncertainty about the future. Psychosocial services should therefore consider both immediate emotional responses to (brady)seismic activity and the cumulative consequences of living under recurrent threat in a bradyseismic area. This approach is consistent with multilevel MHPSS frameworks, which combine community-based support with more intensive and specialized interventions according to the severity and specific nature of psychosocial needs (IASC, 2007, 2021, 2022; Sphere Association, 2018).

### 5.3. Agency and the Preparedness Phase

The co-occurrence of experience, acceptance, and cognitive reappraisal with both preparedness and resignation highlights the importance of strengthening perceived agency. Preparedness initiatives could translate familiarity with bradyseism into actionable competencies through clear plans, practical exercises, and guidance on when and how to adopt protective behaviours. This may help distinguish informed adaptation from reduced behavioural responsiveness or passive acceptance, without assuming that familiarity with earthquakes is equivalent to adequate preparedness. More broadly, integrating psychosocial support into the preparedness phase may help move interventions beyond a predominantly reactive, post-event approach (Gray et al., 2021; IASC, 2021). Moreover, prolonged exposure to environmental risks such as bradyseism tends to blur the conventional distinction among the classical phases of preparedness, response, and recovery, potentially requiring specifically tailored approaches to practice.

### 5.4. Community- and Place-Based Support

The relevance of these community resources is consistent with evidence showing that social support, group involvement, and social infrastructure contribute to social-psychological adaptation following disasters (Aldrich, 2023; Gallagher et al., 2019; Kaniasty, 2020). Psychosocial interventions should therefore be coordinated with community, housing, economic, and institutional support, particularly for residents experiencing displacement or disruption of everyday life.

More broadly, the predominantly cross-quadrant nature of the identified constellations suggests that interventions should not focus on individual resources in isolation but integrate personal, social, and institutional resources (at both general and risk-specific levels). The identified configurations may provide an exploratory basis for developing a matrix linking different psychological outcome profiles to the resources with which they co-occur in the narratives. Following further investigation and validation, such a matrix could assist local authorities in identifying the resources relevant to each profile of social-psychological distress and in planning differentiated, context-sensitive psychosocial interventions integrated with risk communication and emergency preparedness strategies (IASC, 2021; Milani et al., 2026; United Nations Office for Disaster Risk Reduction, 2015; WHO, 2019).

## 6. Limitations

Although this study provides a comprehensive exploration of psychological outcomes and resources in the context of bradyseism, several limitations should be acknowledged. Purposive sampling and the inclusion of participants with different roles and experiences enabled the collection of a diverse range of perspectives, but the findings cannot be considered representative of the population of the *Campi Flegrei*. Moreover, although all participants were inhabitants of the *Campi Flegrei* area, those recruited based on institutional, professional, or community roles could report both personal experiences and observations arising from their roles. These narrative perspectives were not systematically distinguished in the analysis. Because the unit of analysis was the narrative segment, the reported frequencies indicate how often distress-related psychological-outcome content was identified in the corpus, regardless of whether it referred to participants’ own experiences or to observations of other community members. Consequently, these frequencies do not indicate how many participants experienced each form of distress and do not support comparisons across recruitment-role groups.

The findings, therefore, reflect both individual experiences and shared representations of the phenomenon. The use of narrative segments as analytic units also means that multiple segments from the same interview contributed separately to the frequency and co-occurrence estimates. Participants contributing more segments therefore had greater opportunity to influence these estimates, and recurrent patterns within the same interview could be represented more than once. Because Lift was calculated from these segment-level frequencies, its values should be interpreted cautiously and intended as descriptive of the coded corpus, not as estimates of associations at the participant level.

A further limitation concerns the retrospective nature of some accounts, in which participants reconstructed experiences of previous bradyseismic crises or earthquakes that occurred at different times. These reconstructions may have been influenced by the time elapsed, subsequent exposure to the phenomenon, and information circulating within the social and media environment. Longitudinal studies conducted during different phases of bradyseismic activity could further examine how distress, threat perception, and resource mobilization change over time. A further methodological consideration concerns the partial semantic overlap between negative emotion and threat and obstacle perception and the distress categories. This overlap may contribute to the observed co-occurrences and corresponding Lift values, which should therefore be interpreted in light of the shared semantic content of these categories.

Finally, co-occurrences describe the joint presence of resources and psychological outcomes within the same analytic unit but do not establish the direction of these associations or demonstrate a buffering function. The identified configurations should therefore be regarded as exploratory hypotheses requiring further investigation through quantitative research, including examining which resources are associated with different psychological outcome profiles and the conditions under which they may serve an adaptive function. The geological, social, cultural and institutional specificity of the *Campi Flegrei* also warrants caution when transferring these findings to other contexts of prolonged environmental threat.

## 7. Conclusions

This study examined manifestations of psychological outcomes (both positive and negative), personal and social resources, and their co-occurrence configurations in the context of prolonged exposure to bradyseism in the *Campi Flegrei*. The narratives reveal distress that extends beyond individual earthquakes and becomes embedded in everyday life through anxiety, hypervigilance, sleep disturbances, demoralization, and a persistent sense of threat. At the same time, experience, acceptance, cognitive reappraisal, risk perception, place attachment, and informational and social resources recur alongside different ways of understanding and managing the phenomenon.

The study’s main contribution lies in reconstructing the configurations through which resources and psychological outcomes coexist amid prolonged environmental threat and in using the SARA model to map available and unavailable resources across four predefined quadrants formed by the intersection of two dimensions: generic versus threat-specific and personal versus social. Some configurations suggest possible pathways through which resources may modulate responses to the threat, without providing direct evidence of a buffering or compensatory function. Other configurations highlight the ambivalence of resources such as risk perception and place attachment, which are associated with both continuities in everyday life and forms of hypervigilance, habituation, or resignation. These findings provide an exploratory basis for subsequent quantitative investigation and for developing a matrix linking specific psychological outcome profiles to the resources with which they co-occur in the narratives, thereby informing the design of differentiated and context-sensitive psychosocial interventions.

## Figures and Tables

**Table 1:** Selected quotations illustrating anxiety-related narratives.

Theme	Participant	Verbatim Quotation
Future uncertainty	P4	“... at the same time, there remains a strong sense of concern and uncertainty about the future...”
	P6	“I can’t tell you what will happen in the future.”
	P18	“... uncertainty about the future [...] a sense of fear and anxiety that prevents [...] living everyday life peacefully.”
Persistent apprehension and vulnerability	P18	“... we live in this constant state of vulnerability and insecurity.”
	P31	“... this constant apprehension has understandably developed and now accompanies everyone’s daily life.”
	P37	“We all live with this anxiety. I can feel it under my skin.”
Constant state of alert and sensitivity to environmental cues	P6	“... you simply can’t tolerate even the slightest tremor...”
	P8	“... you always reach a state of constant alertness...”
	P10	“... we are always on alert because we are frightened.”
	P17	“I know people who have never unpacked their emergency bag or who always keep their car’s fuel tank full in case they have to flee.”
	P50	“... we live with the tension of the unknown, of not knowing what will happen [...] we often startle [...] especially at night.”
	P52	“... even when a truck passes by, the first reaction is fear... On this road, every time a heavy vehicle goes past, people worry that it is another earthquake. At the beginning, it was terrible; you felt completely lost and kept asking yourself, ‘What are we heading towards?’”

**Table 2:** Selected quotations illustrating post-traumatic stress-related narratives.

Theme	Participant	Verbatim Quotation
Intense seismic events as enduring narrative reference points	P31	“... from that night onwards, the population entered a state of terror...”
	P35	“... the magnitude 4.4 earthquake was terrifying... we felt as though we had looked death in the face...”
Persistent loss of safety and avoidance of the home	P29	“... I’m not going back home alone... I would rather remain sitting on a chair than go back home...”
	P30	“... the house had been declared habitable... but he did not want to return...”
Intense emotional reactivity and context-dependent vulnerability	P17	“... for those who had never experienced an earthquake, it was something terrifying...”
	P17	“... being confined during an earthquake is a terrifying experience... it is like having the floor disappear beneath your feet, but from behind a locked door...”
	P36	“... we saw children from our parish vomiting from fear...”

**Table 3:** Selected quotations illustrating depression-related narratives.

Theme	Participant	Verbatim Quotation
Loss of hope and resignation about the future	P4	“... bradyseism keeps recurring... they are somewhat resigned... there remains a strong concern and uncertainty about the future... some people are leaving the area, while others are being pushed out of employment...”
Collective demoralization and territorial decline	P6	“... residents say, ‘Pozzuoli is dead now’... on the other hand, there is a desire to receive answers... rather than leaving, I would like greater protection.”
	P31	“... it is as though we had been assigned this inescapable destiny... economic depression, businesses closing, people leaving their homes...”
Sadness associated with the Normalization of threat	P45	“It is somewhat sad... because it is sad to become accustomed to an earthquake... it is difficult to get used to all this, but I think the population has become somewhat accustomed to it.”

**Table 4:** Selected quotations illustrating sleep-related narratives.

Theme	Participant	Verbatim Quotation
Repeated sleep disruption during seismic swarms	P6	“... I came to understand the fear... of those who cannot sleep, of those who sleep in their cars...”
	P11	“... for us, it means not sleeping for several consecutive nights and remaining in a state of constant alertness...”
	P51	“... nothing happens for a week, and then you do not sleep at night for four days.”
Changes in sleeping arrangements	P28	“... people continued to sleep in their cars...”
	P29	“... I would rather remain sitting on a chair than go back home... during the day they stayed at home, but at night they came here to sleep...”
Nocturnal vigilance and anticipation of renewed seismic activity	P45	“... I know what it means to sleep badly at night because you do not know whether you will suddenly be awakened by an earthquake...”
	P50	“... we live with the tension of the unknown... we often startle during the night...”

**Table 5:** Selected quotations illustrating post-traumatic growth-related narratives.

Theme	Participant	Verbatim Quotation
Positive reappraisal of the territory and its identity	P7	“The rumbling sound that precedes earthquakes is an extraordinary experience of contact with nature: experiencing an earthquake under safe conditions could become a cultural initiative representing the essence of the territory.”
	P22	“... we always try to transform difficulty into an opportunity. I am not saying that we should claim that it is good to experience bradyseism at home... but highlighting the volcanic and sulfurous characteristics, and therefore the distinctiveness of a place such as the *Campi Flegrei*, could even become a source of learning, provided that this is embedded within a framework of prevention and safety.”
Community development emerging from adversity	P21	“... bradyseism actually also has positive aspects because, through this difficulty, the territory is gaining greater recognition...”

## Data Availability

The data are not publicly available because the interview transcripts contain potentially identifiable and sensitive information, and their public sharing was not included in the informed consent provided by participants. Further information may be requested from the corresponding author, subject to ethical and privacy restrictions.

## Supplementary Materials

The following supporting information can be downloaded at: https://www.mdpi.com/article/10.3390/ejihpe16100164/s1, S1, Sample Characteristics and Additional Descriptive Findings (Table S1, Sample composition and participant characteristics; Table S2, Habituation-related accounts); S2, Semi-structured Interview Outline; S3, Coding Manual for Distress Clusters (Table S3, Summary table of operational definitions and inclusion criteria for distress); S4, Coding Manual for Personal and Social Resources (Table S4a, Summary table of operational definitions and inclusion criteria for resources; Table S4b, Summary table of operational definitions and inclusion criteria for resource states; Table S5, Example of Coding a Single Interview); S5, Descriptive Statistics for the Coded Corpus and Resource–Outcome Co-occurrences (Table S6, Frequencies of psychological outcome categories across analytic units and participants; Table S7, Frequencies of resource states across analytic units and participants; Table S8, Observed resource-state–psychological outcome co-occurrences and Lift values); S6, Consolidated Criteria for Reporting Qualitative Research (COREQ): 32-item checklist.
