# Supplementary material for: Psychological Distress and Resources Under Prolonged Exposure to an Environmental Threat: A Qualitative Study on Inhabitants Exposed to the Campi Flegrei’s Bradyseism
Source: Eur J Investig Health Psychol Educ. 2026 Oct 14;16(10):164. doi: 10.3390/ejihpe16100164 (PMC13703216; doi:10.3390/ejihpe16100164)
Supplement: Supplementary file 1 [file ejihpe-16-00164-s001.zip › ejihpe-4542075-supplementary.pdf]

SUPPLEMENTARY MATERIALS

These Supplementary Materials accompany the article "Psychological Distress and Resources under Prolonged Exposure to an Environmental Threat: A Qualitative Study on Inhabitants Exposed to the Campi Flegrei's Bradyseism." They are organized into six parts, providing additional information on the research sample, data collection, coding framework, descriptive analyses, and qualitative reporting.

S1. Research Sample Descriptives: This table provides detailed information on the composition and characteristics of the research sample (Table S1) and presents the habituation-related accounts identified in the interview corpus (Table S2).

S2. Semi-structured Interview Outline: This section reports the interview guide used for data collection, including the main questions and optional probes concerning participants' experience of bradyseism and personal and social resources.

S3. Coding Manual for Distress Clusters: This section describes the coding framework used to identify psychological outcome categories, including the coding procedure, unit of analysis, general coding rules, and operational definitions and inclusion criteria for each category (Table S3).

S4. Coding Manual for Personal and Social Resources: This section describes the coding framework used to identify SARA-derived personal and social resources and their states. It provides the operational definitions and inclusion criteria for the resource categories (Table S4a) and resource states (Table S4b), together with examples illustrating the application of the coding scheme to interview excerpts (Table S5).

S5. Descriptive Statistics for the Coded Corpus and Resource–Outcome Co-occurrences: This section provides the descriptive statistics underlying the analyses reported in the main text, including frequencies of psychological outcome categories across analytic units and participants (Table S6), frequencies of resource states across analytic units and participants (Table S7), and observed resource-state–psychological outcome co-occurrences and Lift values (Table S8).

S6. Consolidated Criteria for Reporting Qualitative Research (COREQ): This 32-item checklist provides the completed COREQ checklist documenting the reporting of the qualitative study.

S1. Sample Characteristics and Additional Descriptive Findings

Table S1. Sample composition and participant characteristics.

P	Age	Gender	Stakeholder_type	Type
P1	50-60	Man	Healthcare professionals (r.n.i.)*	Napoli
P2	50-60	Man	Institutional representatives	Pozzuoli
P3	50-60	Woman	Institutional representatives	Bacoli
P4	40-50	Woman	Profit association	Bacoli
P5	40-50	Woman	Education sector	Pozzuoli
P6	30-40	Woman	Community members*	Pozzuoli
P7	40-50	Woman	Institutional representatives	Pozzuoli
P8	30-40	Woman	Healthcare professionals (r.n.i.)*	Pozzuoli
P9	30-40	Man	Profit association	Pozzuoli
P10	30-40	Man	Community members	Quarto

P11	30-40	Woman	Healthcare professionals (r.i.)*	Pozzuoli
P12	50-60	Woman	Profit association	Pozzuoli
P13	50-60	Woman	Profit association	Pozzuoli
P14	over 60	Man	Nonprofit association	Pozzuoli
P15	over 60	Man	Nonprofit association	Pozzuoli
P16	40-50	Man	Nonprofit association (r.n.i)*	Napoli
P17	30-40	Woman	Nonprofit association	Pozzuoli
P18	30-40	Woman	Profit association	Bacoli
P19	40-50	Woman	Nonprofit association	Quarto
P20	30-40	Woman	Law enforcement	Pozzuoli
P21	30-40	Woman	Nonprofit association	Pozzuoli
P22	50-60	Man	Profit association	Pozzuoli
P23	30-40	Man	Nonprofit association	Bacoli
P24	30-40	Man	Nonprofit association	Bacoli
P25	30-40	Man	Nonprofit association	Bacoli
P26	30-40	Man	Nonprofit association	Bacoli
P27	30-40	Man	Nonprofit association	Bacoli
P28	30-40	Man	Community members	Pozzuoli
P29	50-60	Man	Nonprofit association	Pozzuoli
P30	50-60	Man	Nonprofit association	Pozzuoli
P31	40-50	Man	Law enforcement	Pozzuoli
P32	50-60	Man	Community members	Pozzuoli
P33	50-60	Man	Religious institution	Pozzuoli
P34	20-30	Woman	Community members	Pozzuoli
P35	20-30	Man	Community members	Pozzuoli
P36	20-30	Woman	Community members	Pozzuoli
P37	20-30	Man	Community members	Pozzuoli
P38	20-30	Woman	Community members	Pozzuoli
P39	20-30	Man	Community members	Pozzuoli
P40	20-30	Woman	Community members	Pozzuoli
P41	20-30	Man	Community members	Pozzuoli
P42	20-30	Man	Community members	Pozzuoli
P43	20-30	Woman	Community members	Pozzuoli
P44	20-30	Man	Community members	Pozzuoli
P45	50-60	Man	Firefighters (r.i.)*	Napoli
P46	50-60	Man	Firefighters (r.i.) *	Napoli
P47	50-60	Man	Firefighters (r.i.)*	Napoli
P48	50-60	Man	Firefighters (r.i.)*	Napoli
P49	30-40	Man	Community members	Bagnoli
P50	30-40	Man	Community members	Bagnoli

P51	50-60	Man	Community members	Pozzuoli
P52	50-60	Woman	Education sector	Bacoli

Note. * (r.n.i.) = rescuer non-institutional; (r.i.) = rescuer institutional. P refers to the participant identification number; "*Community members*" identifies participants recruited in their capacity as inhabitants; and the remaining categories indicate the professional, institutional, or community role through which participants were recruited. Residence in the Campi Flegrei area was an eligibility criterion for all participants.

Table S2. Habituation-related accounts.

Participant	Description of the response or related process	Explicit reference to habituation
P27	"When we felt the first tremors, I obviously reacted more strongly, but much less so over time. During some periods, they are felt every day, even several times a day, so I tend to react less as time passes."	
P23		"Anyway, in my opinion, they [the residents of Pozzuoli] are even more accustomed to it."
P24	"In my case, I experience it as frightening, yes, but then it passes as if it were—I don't know—rain, let's put it that way."	"In some respects, we are 'accustomed' to it."
P33	"Psychologically, by now, let's say, we have become somewhat accustomed to it. And so, as I said before, we no longer go downstairs. We only go downstairs from magnitude 4 onward. Previously, we would already go downstairs at 2.5, but not anymore."	"We have become somewhat accustomed to it."
P40	"Personally, however, I have become accustomed to it by now; I am no longer afraid as I was at first. So, even when they occur at night—for example, when [the tremor] recently occurred at night—my mother and father went downstairs and sat in the car, while I stayed at home and slept in bed."	"I have become accustomed to it by now."
P31	"At this stage, there is a certain habituation, but only up to a tolerance threshold. Generally, when [the magnitude] exceeds 3.5, it becomes chaos. Below 3.5, it no longer really counts, so there is some habituation, but it is a depressed situation."	"Habituation"; "habituated."
P45	"Although it is a little sad to say, people have become somewhat accustomed to bradyseism. We can also see this from the number of calls we receive—we now receive fewer calls to the emergency switchboard. By now, people have some idea of how to behave following earthquakes and have also become accustomed to them. This is a little sad, because it is sad to become accustomed to an earthquake."	"People have become somewhat accustomed to bradyseism."

P10		"We are a little more accustomed to it."
P46		"It is as if people have become accustomed to it. [With magnitudes of] 2.3 or 2.1, they live with it; it was so unusual, but people become accustomed to it."
P2		"I would not say that the population is accustomed to the phenomenon, but it lives with it."
P16	"In my opinion, risk perception has changed. In what sense? It has weakened somewhat... It is as if people have begun to live with that fear of the tremors, so those tremors have become part of their everyday lives."	
P17		"I live with it and I am accustomed to it. I do not live with an atavistic terror; when a tremor occurs, I do not run away—at most, I take shelter under the table."
P18	"...You are considered 'accustomed' when they tell me, 'Well, you're accustomed to it, aren't you?' It is a bit of a double-edged phrase, because yes, being accustomed in the sense of being trained: I recognize that it may be a tremor, so I am trained in the sense that I know how to behave. But it is not really something you become accustomed to."	
P19		"We have become accustomed to it."
P22		"As time passes, people are beginning to become accustomed to these phenomena."

S2. Semi-structured Interview Outline

Area 1. Experience of Living with the Phenomenon of Bradyseism

Input Question

Can you tell me how you're coping with the bradyseism phenomenon in the Phlegraean Fields?

Feedback

• What has changed most in your daily life?
• Which aspects of the situation do you consider most significant?
• Are there any episodes you particularly remember?

Area 2. Personal and Social Resources

Input Question

Reflecting on your experience, what has helped you most in dealing with this situation of uncertainty?

Feedback

- What would most help you adapt?
- Are there any personal or social resources that you've lacked?
- What makes dealing with this situation more difficult?
- What role do institutions play?

S3. Coding Manual for Distress Clusters

S3.1. Background

This coding manual is designed to identify the main clusters of psychological distress within a corpus of qualitative interviews collected in the *Campi Flegrei* area as part of the RETURN project—*Multi-risk Science for Resilient Communities under a Changing Climate*.
The distress clusters included in this manual were derived from the findings of a systematic review of the literature on the psychological impacts of geophysical natural disasters (e.g., earthquakes, volcanic activity, and landslides).
The natural disaster literature consistently shows that exposure to geophysical events is associated with a recurring range of adverse psychological outcomes, including:

- Post-traumatic stress symptoms (PTSS);
- Anxiety and states of hyperarousal;
- Depression and feelings of helplessness;
- Sleep disturbances.

Exposure to geophysical events is also associated with impaired general psychological well-being (Norris et al., 2002; Goldmann & Galea, 2014; Lowe et al., 2019).
Alongside these adverse outcomes, some studies have documented the occurrence of post-traumatic growth (PTG), defined as the development of new personal, relational, and existential resources following the experience of a critical event (Tedeschi & Calhoun, 2004).

S3.2. Coding Procedure

The coding process aims to identify distress clusters, defined based on the natural disaster literature, using explicit, replicable operational criteria.
The coding procedure involves:
- Carefully reading each of the following text excerpts;
- Assigning the code based on its overall meaning and on explicit linguistic markers;
- Recording the presence or absence of each cluster:
  - 1 = present;
  - 0 = absent.

The coding procedure is applied consistently across all interviews.

S3.3. Unit of Analysis and General Rules

The unit of analysis consists of a paragraph drawn from the verbatim transcript.

Coding rules
Coding is based exclusively on the explicit content of the text.

- The same excerpt may be assigned to multiple clusters.
- In cases of uncertainty, no code should be assigned, in accordance with a conservative coding criterion.

S3.4. Structure of the Coding System

Coding focuses exclusively on the following distress clusters:

- Post-traumatic stress symptoms (PTSS);
- Anxiety;
- Depression;
- Sleep disturbances;
- Suicidal thoughts;
- Post-traumatic growth (PTG).

The table below (Table S3) provides an operational definition of each cluster. Please read it carefully before starting coding. Please refer to the provided linguistic markers as supplementary inclusion criteria.

Table S3. Summary of operational definitions and inclusion criteria for distress.

Cluster	Operational definition	Inclusion criteria (linguistic markers)
Post-traumatic stress symptoms (PTSS)*	Intense reactions associated with a traumatic experience or emotional reactivation: intrusive experiences, avoidance, heightened sensitivity, and hypervigilance	"traumatic"; "shocking"; "panic"; "intense terror"; "reliving" past events; "vomiting"; "paralysis"
Anxiety*	A persistent state of arousal, worry, or negative anticipation	"anxiety"; "worry"; "apprehension"; "agitation"; anticipation of an earthquake tremor; hypervigilance
Depression	Low mood or feelings of helplessness/resignation, and having to live with the threat/risk	"depression"; "depressed"; a sense of inevitable fate (when explicitly expressed); resignation; and a sense of helplessness
Sleep disturbances	Sleep disturbances related to the threat	"sleeping poorly"; "unable to sleep"; "night-time is the worst time"; sleeping away from home/in a car
Suicidal thoughts	Explicit references to suicidal ideation	any direct reference to suicidal ideation
Post-traumatic growth (PTG)	Positive changes resulting from the experience of the threat	greater personal strength; new meanings; relational/existential growth

Hypervigilance-related content was coded under both categories because it may occur within both domains. Anxiety captured persistent arousal, worry, and negative anticipation, whereas PTSS captured reactions framed in relation to a traumatic experience or its emotional reactivation. These distress categories are neither mutually exclusive nor intended to provide an exhaustive classification of distress.

S3.5. Final Coding Guidelines

Coding must be applied systematically and consistently across all excerpts of each interview.
The same excerpt may be assigned to multiple clusters when it contains markers associated with more than one psychological outcome.

S4. Coding Manual for Personal and Social Resources

S4.1. Background

The thematic analysis was conducted on a corpus of qualitative interviews collected as part of the RETURN project—*Multi-risk Science for Resilient Communities under a Changing Climate*—an extended partnership funded by Italy's National Recovery and Resilience Plan (PNRR). The project aims to investigate environmental risks and the conditions that foster resilience among communities exposed to multi-risk scenarios.
As part of the RETURN activities, a collaboration was established with the Italian Civil Protection Department (DPC) to investigate the psychosocial implications of bradyseism in the *Campi Flegrei* area. The activity addresses a context of chronic risk and examines the sociocultural and communication processes that shape the relationship between institutions and the local population, with particular attention to interventions targeting local residents.
The interviews were coded according to the SARA model (*Social-psychological Antecedents to Resilience for Adaptation*), a conceptual framework that organizes the main psychosocial predictors of adaptation across the preparedness, response, and recovery phases. The model distinguishes between personal and social resources, considering both their general dimension and their specificity to the risk.

S4.2. Coding Procedure

The coding task aims to record the status (available, unavailable, or not identified) of the SARA-derived resources based on what is explicitly reported in the interview transcripts.
The coding procedure involves:

- Carefully reading each of the following text excerpts;
- Assigning the code based on explicit linguistic markers;
- Recording the state of each resource category.

The coding procedure is applied consistently across all interviews.

S4.3. Unit of Analysis and General Rules

The unit of analysis consists of a text excerpt corresponding to a paragraph drawn from the verbatim interview transcripts.
Coding is based solely on the explicit content of the text.
The same text excerpt may be associated with multiple psychosocial resource categories. Each resource is coded separately, and its corresponding status—available, unavailable, or not identified—is recorded.
When a resource is not mentioned in the participant's account, it is recorded as not identified.

S4.4. Structure of the Coding System

The coding system is based on the SARA conceptual framework and includes 29 psychosocial resources relevant to adaptation across the disaster cycle. These resources constitute the predefined analytical categories against which all interview excerpts are coded.
The coding procedure combines two dimensions:

1. **Resource type**, corresponding to one of the 29 resources included in the SARA framework.
2. **Resource status**, indicating whether each resource is available, unavailable, or not identified in the excerpt.

For each excerpt, coders assess all 29 resources and assign a status to each. The same excerpt may contain evidence concerning multiple resources. Accordingly, more than one resource may be coded as available or unavailable within the same excerpt. Resources for which no explicit evidence is found are coded as not identified.
The complete list of resources, together with their operational definitions and inclusion criteria, is provided in Table S4a. The complete list of resource states, along with their operational definitions and inclusion criteria, is provided in Table S4b.

Table S4a. Summary of operational definitions and inclusion criteria for resources.

Resource	Conceptual definition	Inclusion criteria

Acceptance coping	Acceptance of the risk situation as part of reality.	Code when the interviewee expresses adaptation to or acceptance of the situation without attempting to change it.
Attitude	Personal evaluation of actions, decisions, or behaviors related to the risk situation.	Code when the interviewee expresses a judgment, opinion, or favorable or unfavorable evaluation of actions, decisions, or behaviors related to the risk situation.
Climate change belief	Belief in the reality of climate change.	Code when the interviewee affirms or denies the existence of climate change.
Descriptive norms	Perceptions of the behaviors actually adopted by others in relation to the risk situation.	Code when the interviewee describes what they observe other individuals, groups, or community members doing to address the risk situation.
Experiences	Direct or indirect experience gained through exposure to previous risk events related to bradyseism or to situations perceived as similar.	Code when the interviewee refers to past events experienced directly or indirectly (e.g., at the personal, family, or community level) that are related to bradyseism or similar situations.
Information	Access to and understanding of risk information.	Code when the interviewee comments on the clarity, reliability, or timeliness of the information received.
Injunctive norms	Perceptions of social approval or disapproval regarding the behaviors to adopt in relation to the risk situation.	Code when the interviewee refers to what is socially approved or disapproved of, or expected or not expected, in addressing the risk situation.
Knowledge	Knowledge of the phenomenon, the associated risks, and appropriate response and protective measures.	Code when the interviewee demonstrates knowledge of the phenomenon, the associated risks, appropriate response or protective measures, or how to access information.
Media influence	Influence exerted by traditional and digital media through exposure to information, alerts, recommendations, or guidance concerning the risk situation.	Code when the interviewee refers to the influence of television, newspapers, radio, websites, social media, or other media channels in shaping perceptions, emotions, decisions, or behaviors related to the risk situation.
Negative emotion	Transient emotions associated with the risk context (e.g., fear or worry). Negative emotion (adapted from negative affect; Tan et al., 2024): negative emotional responses to natural hazards, including fear, depression, anxiety, and worry.	Code when negative emotional experiences emerge in relation to the risk situation.
Optimism	Positive expectations regarding the future and the outcomes of the risk situation.	Code when the interviewee expresses confidence or hope regarding future improvement or favorable outcomes.
Outcome efficacy	Belief that protective actions are effective in reducing risk.	Code when the interviewee evaluates the practical usefulness of the actions taken to mitigate and/or reduce the risk.
Place attachment	An emotional and identity-based bond with a place.	Code when the interviewee expresses attachment to the local area.

Quality of life	Perception of one's general well-being and living conditions within one's life context.	Code when the interviewee refers to their well-being, quality of life, or living conditions that they consider important to preserve or perceive as being adversely affected by the context.
Reappraisal coping	A cognitive emotion-regulation strategy aimed at changing how emotionally stressful situations are evaluated and modifying their impact to make them more manageable.	Code when the interviewee reinterprets a stressful situation, reframing an event perceived as threatening as a manageable challenge.
Religious coping	Use of faith or religious practices as an adaptation resource.	Code when the interviewee mentions prayer, religious faith, or reliance on God as a form of support.
Responsibility	Sense of personal responsibility for protecting oneself or others in relation to the risk context.	Code when the interviewee expresses a personal or family duty to act in relation to the risk context.
Risk perception	Subjective perception of the dangerousness, likelihood, and/or severity of the possible consequences associated with bradyseism.	Code when the interviewee evaluates the phenomenon in terms of dangerousness, likelihood of occurrence, and/or severity of its possible consequences.
Self-efficacy	Confidence in one's ability to effectively address risk situations and adopt protective behaviors.	Code when the interviewee expresses confidence or lack of confidence in their ability to prepare for, respond to, or manage the consequences of a critical event.
Sense of community	Sense of belonging to and cohesion with the community.	Code when the interviewee refers to solidarity, cooperation, or community ties.
Social cues	Observable environmental and social cues related to the risk situation.	Code when the interviewee refers to observable environmental or social cues associated with the risk situation (e.g., observed conditions of the phenomenon, closure of businesses, evacuation of other people, or observable changes in the local context).
Social support	Emotional, informational, or practical support is perceived as available from other people.	Code when the interviewee mentions support received from or available through others.
Spirituality	A spiritual orientation or search for meaning that is not necessarily related to religious practices.	Code when the interviewee refers to spiritual or inner dimensions, or sources of meaning, used to cope with the risk.
Subjective norms	Perceptions of what others generally approve or disapprove of.	Code when the interviewee reports what they believe others approve or disapprove of.
Support coping	A coping strategy based on actively seeking social support to address the risk situation and manage uncertainty or distress.	Code when the interviewee describes actively turning to other individuals, groups, or institutions to obtain support, information, guidance, or assistance in managing the risk situation.
Threat and obstacle perception	Assessment of the perceived threat and barriers to protective action. Threat and obstacle perception (adapted from threat and obstacle; Tan et al., 2024): perceived threats and obstacles to protective action.	Code when the interviewee describes perceived difficulties, costs, or barriers.
Trust in government	General trust in institutions and their ability to manage the risk situation.	Code when the interviewee expresses trust or distrust in institutions or authorities.

Trust in official orders	Trust in the operational instructions provided by the authorities.	Code when the interviewee expresses trust or distrust in official instructions.
Trust in the measures implemented	Trust in the effectiveness of the protective measures implemented for the risk situation.	Code when the interviewee evaluates positively or negatively the measures implemented in relation to the risk situation.

Table S4b. Summary of operational definitions and inclusion criteria for resource states

Resource State	Conceptual definition	Inclusion criteria
Available	The resource is recognized and activated in the participant's account and is described as present and usable within the relevant context.	The "*available*" status is assigned whenever an interviewee provides evidence of owning, using, or drawing support from a resource, or narrates actions that demonstrate its practical application.
Unavailable	The specific resource is acknowledged but narrated as being deficient, restricted, or lacking accessibility within the framework of the participant's experience.	The "*unavailable*" status is assigned whenever the participant's account indicates that a resource is inaccessible or insufficient, or when they explicitly report failing to receive an effective response to a recognized need.
Not identified	The resource is neither recognized nor activated in the participant's account and does not appear in the transcripts as an explicitly mentioned element.	The '*not identified*' status is assigned if the textual material fails to offer manifest or unequivocal indicators regarding the presence or absence of a specific resource.

An example of applying the coding scheme to interview excerpts is reported in Table S5, illustrating the coding of both resources and resource states.

Table S5. Example of coding a single interview.

Resource	Verbatim quotation	Resource state
Negative emotion	"There, I had the sense—and also the fear—that I could lose everything. "Such strong tremors then put you in a state of anxiety." (P6)	Available<br><br>Available
Risk perception	"Then there was one of the strongest tremors, the 4.5 one." (P6)	Available
Place attachment	"It is really difficult to imagine that, at some point, you take everything and leave." (P6)<br><br>"I have a deep, visceral love for the *Campi Flegrei*." (P6)	Available<br><br>Available
Information	"I have also seen a lot of incorrect information." (P6)	Unavailable
Media influence	"There have been newspapers and television news programs with catastrophic reports." (P6)	Unavailable
Trust in government	"I would like more attention from those who govern us." (P6)	Unavailable
Social cues	"There were people close to me who left." (P6)	Available
Knowledge	"In schools, they taught [children] to get under their desks and to know how to escape." (P6)	Available

The coding procedure is applied separately to each excerpt and involves the following steps:

- Read the complete excerpt and consider its meaning within the relevant interview context.
- Assess the excerpt against each of the 29 resources included in the SARA framework.
- For each resource, determine whether the excerpt provides explicit evidence that the resource is available, unavailable, or not identified.
- Assign one of the following mutually exclusive status codes to each resource:

a. Available: 1;
b. Unavailable: −1;
c. Not identified: 0.

- Record the excerpt as supporting evidence for each resource coded as available or unavailable.

Coding decisions must be based exclusively on the explicit content of the interview transcripts. Coders should not infer the presence, absence, effectiveness, or accessibility of a resource from information that is not directly stated or clearly expressed in the excerpt. In cases of uncertainty, the resource should be coded as not identified, in accordance with a conservative coding criterion.

S4.5. Final Coding Guidelines

Coding must be applied systematically and consistently across all interviews, following the structure, definitions, and criteria set out in this manual.
The same text excerpt may be assigned to multiple resource categories when it refers to more than one resource. In such cases, each resource must be coded separately and its corresponding status recorded.

S5. Descriptive Statistics for the Coded Corpus and Resource-Outcome Co-occurrences

Tables S6, S7, and S8 provide the descriptive statistics underlying the analyses reported in the main text. Table S6 reports psychological outcome frequencies at the analytic unit and participant levels, Table S7 reports the corresponding frequencies for resource states, and Table S8 reports observed resource-state co-occurrences with outcome, participant counts, and Lift values.

Table S6. Frequencies of psychological outcome categories across analytic units and participants.

Psychological outcome	Analytic units, *n*	Analytic units, %	Participants, *n*
Post-traumatic stress-related experiences	18	10.97	14
Anxiety	54	32.93	30
Depression-related experiences	12	7.32	11
Sleep disturbances	9	5.49	8
Suicidal ideation	0	0.0	0
Post-traumatic growth	4	2.44	4

Note. Analytic unit counts indicate the number of the 164 coded analytic units in which each psychological outcome was identified. Participant counts indicate the number of unique participants contributing at least one analytic unit coded for the corresponding outcome. Categories were not mutually exclusive; therefore, percentages do not sum to 100%. No analytic unit was coded for suicidal ideation.

Table S7. Frequencies of resource states across analytic units and participants.

SARA domain	Resource	State	Analytic units, n	Analytic units, %	Participants, n
Personal–generic	Acceptance coping	Available	47	28.66	30
Personal–generic	Acceptance coping	Unavailable	0	0.00	0
Personal–generic	Optimism	Available	7	4.27	6
Personal–generic	Optimism	Unavailable	1	0.61	1
Personal–generic	Quality of life	Available	0	0.00	0
Personal–generic	Quality of life	Unavailable	5	3.05	4
Personal–generic	Reappraisal coping	Available	23	14.02	15
Personal–generic	Reappraisal coping	Unavailable	0	0.00	0
Personal–generic	Religious coping	Available	3	1.83	3
Personal–generic	Religious coping	Unavailable	0	0.00	0
Personal–generic	Self-efficacy	Available	4	2.44	3
Personal–generic	Self-efficacy	Unavailable	1	0.61	1
Personal–generic	Spirituality	Available	0	0.00	0
Personal–generic	Spirituality	Unavailable	0	0.00	0
Personal–risk-specific	Attitude	Available	0	0.00	0
Personal–risk-specific	Attitude	Unavailable	0	0.00	0
Personal–risk-specific	Climate change belief	Available	0	0.00	0
Personal–risk-specific	Climate change belief	Unavailable	0	0.00	0
Personal–risk-specific	Experiences	Available	21	12.80	15
Personal–risk-specific	Experiences	Unavailable	2	1.22	1
Personal–risk-specific	Information	Available	5	3.05	4

Personal–risk-specific	Information	Unavailable	23	14.02	14
Personal–risk-specific	Knowledge	Available	18	10.98	14
Personal–risk-specific	Knowledge	Unavailable	2	1.22	2
Personal–risk-specific	Negative emotion	Available	37	22.56	23
Personal–risk-specific	Negative emotion	Unavailable	0	0.00	0
Personal–risk-specific	Outcome efficacy	Available	12	7.32	8
Personal–risk-specific	Outcome efficacy	Unavailable	2	1.22	2
Personal–risk-specific	Responsibility	Available	2	1.22	1
Personal–risk-specific	Responsibility	Unavailable	2	1.22	2
Personal–risk-specific	Risk perception	Available	39	23.78	26
Personal–risk-specific	Risk perception	Unavailable	20	12.20	16
Personal–risk-specific	Threat and obstacle perception	Available	20	12.20	16
Personal–risk-specific	Threat and obstacle perception	Unavailable	0	0.00	0
Social–generic	Place attachment	Available	34	20.73	23
Social–generic	Place attachment	Unavailable	0	0.00	0
Social–generic	Sense of community	Available	11	6.71	10
Social–generic	Sense of community	Unavailable	0	0.00	0
Social–generic	Social support	Available	1	0.61	1
Social–generic	Social support	Unavailable	1	0.61	1
Social–generic	Subjective norms	Available	1	0.61	1
Social–generic	Subjective norms	Unavailable	0	0.00	0
Social–generic	Support coping	Available	7	4.27	6
Social–generic	Support coping	Unavailable	1	0.61	1
Social–risk-specific	Descriptive norms	Available	1	0.61	1

Social–risk-specific	Descriptive norms	Unavailable	0	0.00	0
Social–risk-specific	Injunctive norms	Available	0	0.00	0
Social–risk-specific	Injunctive norms	Unavailable	0	0.00	0
Social–risk-specific	Media influence	Available	1	0.61	1
Social–risk-specific	Media influence	Unavailable	11	6.71	10
Social–risk-specific	Social cues	Available	8	4.88	7
Social–risk-specific	Social cues	Unavailable	0	0.00	0
Social–risk-specific	Trust in government	Available	5	3.05	4
Social–risk-specific	Trust in government	Unavailable	23	14.02	16
Social–risk-specific	Trust in measures implemented	Available	6	3.66	4
Social–risk-specific	Trust in measures implemented	Unavailable	12	7.32	8
Social–risk-specific	Trust in official orders	Available	0	0.00	0
Social–risk-specific	Trust in official orders	Unavailable	3	1.83	3

Note. Analytic unit counts indicate the number of the 164 coded analytic units in which each resource state was identified. Participant counts indicate the number of unique participants contributing at least one analytic unit containing that resource state. Available and unavailable states were mutually exclusive for a given resource within an analytic unit, whereas multiple different resources could be coded within the same unit. Percentages are calculated relative to the 164 analytic units.

Table S8. Observed resource-state–psychological outcome co-occurrences and Lift values.

SARA domain	Resource	State	Psychological outcome	Resource-state units, n	Outcome units, n	Co-occurring units, n	Participants, n	Lift
Personal–generic	Acceptance coping	Available	Anxiety	47	54	20	16	1.29
Personal–generic	Acceptance coping	Available	Post-traumatic stress-related experiences	47	18	4	4	0.78
Personal–generic	Acceptance coping	Available	Depression-related experiences	47	12	7	6	2.04
Personal–generic	Acceptance coping	Available	Sleep disturbances	47	9	1	1	0.39
Personal–generic	Acceptance coping	Available	Post-traumatic growth	47	4	1	1	0.87
Personal–generic	Optimism	Available	Post-traumatic growth	7	4	3	3	17.57
Personal–generic	Optimism	Unavailable	Anxiety	1	54	1	1	3.04
Personal–generic	Optimism	Unavailable	Depression-related experiences	1	12	1	1	13.67
Personal–generic	Quality of life	Unavailable	Anxiety	5	54	5	4	3.04
Personal–generic	Reappraisal coping	Available	Anxiety	23	54	10	9	1.32
Personal–generic	Reappraisal coping	Available	Post-traumatic stress-related experiences	23	18	2	2	0.79
Personal–generic	Reappraisal coping	Available	Depression-related experiences	23	12	2	2	1.19
Personal–generic	Reappraisal coping	Available	Post-traumatic growth	23	4	3	3	5.35
Personal–generic	Religious coping	Available	Anxiety	3	54	1	1	1.01
Personal–generic	Religious coping	Available	Post-traumatic stress-related experiences	3	18	2	2	6.07
Personal–generic	Self-efficacy	Available	Anxiety	4	54	1	1	0.76
Personal–risk-specific	Experiences	Available	Anxiety	21	54	7	5	1.01
Personal–risk-specific	Experiences	Available	Post-traumatic stress-related experiences	21	18	4	3	1.74
Personal–risk-	Experiences	Available	Depression-related experiences	21	12	1	1	0.65

specific								
Personal–risk-specific	Experiences	Available	Sleep disturbances	21	9	1	1	0.87
Personal–risk-specific	Experiences	Unavailable	Anxiety	2	54	2	1	3.04
Personal–risk-specific	Experiences	Unavailable	Post-traumatic stress-related experiences	2	18	2	1	9.11
Personal–risk-specific	Information	Available	Anxiety	5	54	1	1	0.61
Personal–risk-specific	Information	Available	Post-traumatic stress-related experiences	5	18	1	1	1.82
Personal–risk-specific	Information	Unavailable	Anxiety	23	54	7	6	0.92
Personal–risk-specific	Information	Unavailable	Post-traumatic stress-related experiences	23	18	3	3	1.19
Personal–risk-specific	Knowledge	Available	Anxiety	18	54	5	4	0.84
Personal–risk-specific	Knowledge	Available	Post-traumatic stress-related experiences	18	18	2	2	1.01
Personal–risk-specific	Knowledge	Available	Depression-related experiences	18	12	1	1	0.76
Personal–risk-specific	Knowledge	Available	Sleep disturbances	18	9	1	1	1.01
Personal–risk-specific	Knowledge	Unavailable	Anxiety	2	54	2	2	3.04
Personal–risk-specific	Negative emotion	Available	Anxiety	37	54	20	14	1.64
Personal–risk-	Negative emotion	Available	Post-traumatic stress-related	37	18	4	4	0.98

specific			experiences					
Personal–risk-specific	Negative emotion	Available	Depression-related experiences	37	12	2	2	0.74
Personal–risk-specific	Negative emotion	Available	Sleep disturbances	37	9	4	4	1.97
Personal–risk-specific	Negative emotion	Available	Post-traumatic growth	37	4	1	1	1.11
Personal–risk-specific	Outcome efficacy	Available	Depression-related experiences	12	12	1	1	1.14
Personal–risk-specific	Outcome efficacy	Unavailable	Post-traumatic stress-related experiences	2	18	1	1	4.56
Personal–risk-specific	Risk perception	Available	Anxiety	39	54	19	15	1.48
Personal–risk-specific	Risk perception	Available	Post-traumatic stress-related experiences	39	18	6	5	1.40
Personal–risk-specific	Risk perception	Available	Depression-related experiences	39	12	5	4	1.75
Personal–risk-specific	Risk perception	Available	Sleep disturbances	39	9	5	4	2.34
Personal–risk-specific	Risk perception	Available	Post-traumatic growth	39	4	1	1	1.05
Personal–risk-specific	Risk perception	Unavailable	Anxiety	20	54	8	7	1.21
Personal–risk-specific	Risk perception	Unavailable	Post-traumatic stress-related experiences	20	18	2	2	0.91
Personal–risk-specific	Risk perception	Unavailable	Depression-related experiences	20	12	3	3	2.05
Personal–risk-	Risk perception	Unavailable	Sleep disturbances	20	9	1	1	0.91

specific								
Personal–risk-specific	Threat and obstacle perception	Available	Anxiety	20	54	5	4	0.76
Personal–risk-specific	Threat and obstacle perception	Available	Post-traumatic stress-related experiences	20	18	2	2	0.91
Personal–risk-specific	Threat and obstacle perception	Available	Sleep disturbances	20	9	1	1	0.91
Social–generic	Place attachment	Available	Anxiety	34	54	10	9	0.89
Social–generic	Place attachment	Available	Post-traumatic stress-related experiences	34	18	4	3	1.07
Social–generic	Place attachment	Available	Depression-related experiences	34	12	5	5	2.01
Social–generic	Place attachment	Available	Sleep disturbances	34	9	1	1	0.54
Social–generic	Place attachment	Available	Post-traumatic growth	34	4	2	2	2.41
Social–generic	Sense of community	Available	Anxiety	11	54	2	2	0.55
Social–generic	Sense of community	Available	Post-traumatic stress-related experiences	11	18	2	2	1.66
Social–generic	Sense of community	Available	Post-traumatic growth	11	4	1	1	3.73
Social–generic	Social support	Available	Anxiety	1	54	1	1	3.04
Social–generic	Social support	Available	Post-traumatic stress-related experiences	1	18	1	1	9.11
Social–generic	Social support	Available	Sleep disturbances	1	9	1	1	18.22
Social–generic	Social support	Unavailable	Anxiety	1	54	1	1	3.04
Social–generic	Subjective norms	Available	Anxiety	1	54	1	1	3.04
Social–generic	Support coping	Available	Anxiety	7	54	2	2	0.87
Social–generic	Support coping	Available	Depression-related experiences	7	12	1	1	1.95
Social–generic	Support coping	Available	Post-traumatic growth	7	4	1	1	5.86
Social–generic	Support coping	Unavailable	Anxiety	1	54	1	1	3.04
Social–risk-specific	Descriptive norms	Available	Anxiety	1	54	1	1	3.04

Social–risk-specific	Media influence	Available	Anxiety	1	54	1	1	3.04
Social–risk-specific	Media influence	Unavailable	Anxiety	11	54	7	7	1.93
Social–risk-specific	Media influence	Unavailable	Post-traumatic stress-related experiences	11	18	1	1	0.83
Social–risk-specific	Social cues	Available	Anxiety	8	54	2	2	0.76
Social–risk-specific	Social cues	Available	Post-traumatic stress-related experiences	8	18	4	4	4.56
Social–risk-specific	Social cues	Available	Depression-related experiences	8	12	1	1	1.71
Social–risk-specific	Social cues	Available	Sleep disturbances	8	9	2	2	4.56
Social–risk-specific	Trust in government	Unavailable	Anxiety	23	54	6	5	0.79
Social–risk-specific	Trust in government	Unavailable	Post-traumatic stress-related experiences	23	18	3	3	1.19
Social–risk-specific	Trust in government	Unavailable	Depression-related experiences	23	12	3	3	1.78
Social–risk-specific	Trust in measures implemented	Unavailable	Anxiety	12	54	2	2	0.51
Social–risk-specific	Trust in measures implemented	Unavailable	Post-traumatic stress-related experiences	12	18	1	1	0.76
Social–risk-specific	Trust in measures implemented	Unavailable	Sleep disturbances	12	9	2	2	3.04
Social–risk-specific	Trust in official orders	Unavailable	Anxiety	3	54	1	1	1.01

Note. Only resource–outcome combinations with at least one observed co-occurrence are reported. Resource-state units and outcome units are the corresponding marginal frequencies across the 164 analytic units. Co-occurring units indicate the number of analytic units in which the resource state and psychological outcome were both coded. Participants indicate the number of unique participants contributing at least one such co-occurring unit. Lift was calculated as the observed co-occurrence divided by the expected co-occurrence, where expected co-occurrence = (resource-state units × outcome units) / 164. Suicidal ideation is not included because no analytic unit was coded for this outcome.

S6. Consolidated criteria for reporting qualitative studies (COREQ): 32-item checklist

Developed from:
Tong A, Sainsbury P, Craig J. Consolidated criteria for reporting qualitative research (COREQ): a 32-item checklist for interviews and focus groups. *International Journal for Quality in Health Care*. 2007. Volume 19, Number 6: pp. 349 – 357.

MANUSCRIPT TITLE: Psychological Distress and Resources under Prolonged Exposure to an Environmental Threat: A Qualitative Study on Inhabitants Exposed to the Campi Flegrei's Bradyseism

**Item No.**	**Guide questions/description**	**Section in which it is reported**
**Domain 1: Research team and reflexivity**		
Personal Characteristics		
1. Interviewer/facilitator	Which author/s conducted the interview or focus group?	Sec. 2.2; Author Contributions
2. Credentials	What were the researcher's credentials? (E.g., PhD, MD)	Sec. 2.2 (psychologists)
3. Occupation	What was their occupation at the time of the study?	Sec. 2.2
4. Gender	Was the researcher male or female?	Supplementary Materials, Table S1
5. Experience and training	What experience or training did the researcher have?	Sec. 2.2
Relationship with participants		
6. Relationship established	*Was a relationship established prior to study commencement?*	Not reported
7. Participant's knowledge of the interviewer	*What did the participants know about the researcher? (e.g., personal goals, reasons for doing the research).*	Sec. 2.1
8. Interviewer characteristics	*What characteristics were reported about the interviewer/facilitator? (e.g., bias, assumptions, reasons, and interests in the research topic)*	Sec. 4, Limitations
**Domain 2: Study design**		
Theoretical framework		
9. Methodological orientation and Theory	*What methodological orientation was stated to underpin the study? (e.g., grounded theory, discourse analysis, ethnography, phenomenology, content analysis).*	Secs. 1.2 and 2.4

Participant selection		
10. Sampling	*How were participants selected? (e.g., purposive, convenience, consecutive, snowball)*	Secs. 2.1 and 2.3
11. Method of approach	*How were participants approached? (e.g., face-to-face, telephone, mail, email)*	Sec. 2.1
12. Sample size	*How many participants were in the study?*	Sec. 2.3 (n = 52)
13. Non-participation	*How many people refused to participate or dropped out? Reasons?*	Not reported
Setting		
14. Setting of data collection	*Where was the data collected? (e.g., home, clinic, workplace)*	Sec. 2.1
15. Presence of non-participants	*Was anyone else present besides the participants and researchers?*	Not reported
16. Description of sample	*What are the important characteristics of the sample? (e.g., demographic data, date)*	Sec. 2.3; Supplementary Materials, Table S1
Data collection		
17. Interview guide	*Were questions, prompts, guides provided by the authors? Was it pilot tested?*	Sec. 2.2; Supplementary Materials S2
18. Repeat interviews	*Were repeat interviews carried out? If yes, how many?*	Not reported
19. Audio/visual recording	*Did the research use audio or visual recording to collect the data?*	Sec. 2.1
20. Field notes	*Were field notes made during and/or after the interview or focus group?*	Not reported
21. Duration	*What was the duration of the interviews or focus group?*	Sec. 2.2 (45-60 min)
22. Data saturation	Was data saturation discussed?	Sec. 2.3 (not a stopping criterion)
23. Transcripts returned	*Were transcripts returned to participants for comment and/or correction?*	Sec. 2.2 (not returned)
**Domain 3: analysis and findings**		
Data analysis		
24. Number of data coders	How many data coders coded the data?	Sec. 2.4 (2 coders on a subset of 16 analytic units for

		intercoder reliability; 1 coder coded the full corpus)

25. Description of the coding tree	*Did the authors provide a description of the coding tree?*	Supplementary Materials S3-S4
26. Derivation of themes	*Were themes identified in advance or derived from the data?*	Sec. 2.4 (a priori); Sec. 3.4 (post hoc)
27. Software	*What software, if applicable, was used to manage the data?*	Sec. 2.5 (Python 3.12)
28. Participant checking	*Did participants provide feedback on the findings?*	Not undertaken
Reporting		
29. Quotations presented	*Were participant quotations presented to illustrate the themes/findings? Was each quotation identified? (e.g., participant number)*	Sec. 3; Tables 1-5
30. Data and findings consistent	*Was there consistency between the data presented and the findings?*	Sec. 3
31. Clarity of major themes	*Were major themes clearly presented in the findings?*	Secs. 3.1-3.3
32. Clarity of minor themes	Is there a description of diverse cases or discussion of minor themes?	Secs. 3.2-3.4

References

Goldmann, E., & Galea, S. (2014). Mental health consequences of disasters. *Annual Review of Public Health*, 35, 169–183. https://doi.org/10.1146/annurev-publhealth-032013-182435

Lowe, S. R., Bonumwezi, J. L., Valdespino-Hayden, Z., & Galea, S. (2019). Posttraumatic stress and depression in the aftermath of environmental disasters: A review of quantitative studies published in 2018. *Current Environmental Health Reports*, 6(4), 344–360. https://doi.org/10.1007/s40572-019-00245-5

Norris, F. H., Friedman, M. J., Watson, P. J., Byrne, C. M., Diaz, E., & Kaniasty, K. (2002). 60,000 disaster victims speak: Part I. An empirical review of the empirical literature, 1981–2001. *Psychiatry*, 65(3), 207–239. https://doi.org/10.1521/psyc.65.3.207.20173

Tedeschi, R. G., & Calhoun, L. G. (2004). Posttraumatic growth: Conceptual foundations and empirical evidence. *Psychological Inquiry*, 15(1), 1–18. https://doi.org/10.1207/S15327965PLI1501_01

Tong, A., Sainsbury, P., & Craig, J. (2007). Consolidated criteria for reporting qualitative research (COREQ): A 32-item checklist for interviews and focus groups. *International Journal for Quality in Health Care, 19*(6), 349–357. https://doi.org/10.1093/intqhc/mzm042
